# Genetic analysis and selection of Bambara groundnut (*Vigna subterranea* [L.] Verdc.) landraces for high yield revealed by qualitative and quantitative traits

**DOI:** 10.1038/s41598-021-87039-8

**Published:** 2021-04-07

**Authors:** Md Mahmudul Hasan Khan, Mohd Y. Rafii, Shairul Izan Ramlee, Mashitah Jusoh, Md Al Mamun

**Affiliations:** 1 Laboratory of Climate-Smart Food Crop Production, Institute of Tropical Agriculture and Food Security (ITAFoS), Universiti Putra Malaysia (UPM), UPM Serdang, 43400 Selangor, Malaysia; 2 Department of Crop Science, Faculty of Agriculture, Universiti Putra Malaysia (UPM), UPM Serdang, 43400 Selangor, Malaysia; 3 Department of Plant Breeding, Bangladesh Agricultural Research Institute (BARI), Gazipur, 1701 Bangladesh; 4 Plant Breeding Division, Bangladesh Jute Research Institute (BJRI), Dhaka, Bangladesh

**Keywords:** Plant breeding, Natural variation in plants, Genetics

## Abstract

As a crop for the new millennium Bambara groundnut (*Vigna subterranea* [L.] Verdc.) considered as leading legumes in the tropical regions due to its versatile advantages. The main intent of this study was to find out the high yielding potential genotypes and considering these genotypes to develop pure lines for commercial cultivation in Malaysia. Considering the 14 qualitative and 27 quantitative traits of fifteen landraces the variation and genetic parameters namely, variability, heritability, genetic advance, characters association, and cluster matrix were determined. ANOVA revealed significant variation for all the agronomic traits (except plant height). Among the accessions, highly significant differences (P ≤ 0.01) were found for almost all the traits excluding fifty percent flowering date, seed length, seed width. The 16 traits out of the 27 quantitative traits had a coefficient of variation (CV) ≥ 20%. A positive and intermediate to perfect highly significant association (r = 0.23 to 1.00; P < 0.00) was found between yield and its related traits. The trait dry seed weight per plant (g) had the highest GCV = 59.91% and PCV = 59.57% whereas the trait fresh pod weight (99.55%), dry seed weight (98.86%), and yield (98.10%) were highly heritable. The genetic advance recorded the highest for dry seed weight (122.01%) and lowest (3.97%) for plant height. To validate the genetic disparity, an unweighted pair-group produce with arithmetic mean (UPGMA), principal component analysis (PCA), heatmap, and H’-index was performed considering 27 quantitative traits. The constructed dendrogram showed five distinct groups of accessions. Genotypes G2, G3, and G9 from Group IV consider as promising lines which gave 70.05% higher mean yield compared to grand mean yield (1180 kg ha^−1^) with desirable traits. Group II had a maximum number of accessions while group III and group V had one of each. However, findings declared that the availability of genetic variance will be beneficial for this crop improvement through cross breeding as well as the plant breeders to prefer desirable traits in *V. subterranea* L. Verdc. for further breeding purposes.

## Introduction

Bambara groundnut is a future emerging legume grown in Africa and Asia, is usually known as a poor man’s crop or as “Women´s Crop”^1^ and newly noted as Crop for New Millennium^2^. The present binomial name *Vigna subterranea* (L.) Verdc was suggested by Verdcourt, 1980^3^ and chromosome number is 2n = 2x = 22^4^. This crop occupied 3^rd^ position after *Arachis hypogea* and *Vigna unguiculate* in African continent^5^ though it is used as food supplement instead of profitable crops due to its low ranked^6^. The word “Bambara’ comes from a place name near Timbuktu in central Mali, West Africa and the word “groundnut’ is the causes of it pods setting occur under the ground soil, hence jointly its common name is ’Bambara groundnut’^7^. Due to rich in carbohydrate (63–65%), protein (18–20%) and oil (17–18%), Bambara groundnut has been defined as a fully well-adjusted food for human feeding^8^. According to^9^ it contains 32.72% essential amino acids and 66.10% non-essential amino acids. The seed is regarded as a balanced food because when compared to other food legumes, it is rich in iron and its protein contains high level of lysine and methionine^10^. Lysine is the major essential amino acid and represents 10.3% of the total essential amino acid^10^. Bambara groundnut can fulfill the regular demand of protein for the marginal users where the animal protein is badly available due to its high cost^11^. Seeds of Bambara groundnut contain considerable amount of minerals such as Ca: 260 mg; K: 1723.25 mg; Fe: 3.6 mg and Na: 75.25 mg of each 100 g dry (weight) seed, moreover due to rich in potassium (K) it also have ability to lessen diabetes by prompting the insulin hormone^12^. The undeveloped fresh seeds of Bambara groundnut can be boiled to make pudding (Moi-Moi or Okpa)^13^; fodders to feed animals^14^ and extract of leaves has medicinal values as anti-vomiting agent^15^. In many developing countries where cultivation of other major crops is difficult, but Bambara groundnut can be accommodated nicely due to its drought tolerant and low diseases-insects infestation nature^16^ and as legume it can fix atmospheric nitrogen via nodulation^17^. This crop has the capacity to give high yield with low input and mostly grown by female in sole culture without any modern techniques^18^ and 10–40% of their total yield they sold in market rest is used by themselves^19^. According to FAOSTAT^20^, the annual production is about 42,023 kg ha^−1^, of which Africa produces half, with Burkina Faso occupied the major producing country. Bambara groundnut can well adapt to the tropical area like Malaysia, where cultivation of major crops (Rice, Wheat, Maize, etc.) are increasingly challenging due to drought and unpredictable rainfall patterns^21^. The average yield of Bambara groundnut is low 0.650–0.850 t ha^−1^^22^, 28.96 g plant^−1^^12^, and 0.38–1.6 t ha^−1^^23^. The production of Bambara groundnut is mainly limited due to lack of improved cultural techniques^24^ and improvement of Bambara groundnut was neglected for many years by researchers because of the lack of available fund and unprivileged effort on its improvement^25^. Although numerous efforts have unsuccessful for the varietal improvement of Bambara groundnut through hybridization due to cleistogamous and autogamous nature of flower and knowledge gap on its reproductive biology as under developing crop^26^. On the aspect of Malaysia, the breeding approach of Bambara groundnut is undetermined, and no commercial high yielding variety is available, so the requirement is to discover commercially high yielding cultivar for certain rising areas^27^. Germplasm screening considering the agronomic variables is the initial attempt to identify the targeted characters of interest^28^. This current research reveals the genetic divergence of fifteen Bambara groundnut accessions to discover the existing variation and the selection to develop high yielding pure lines for this crop improvement. Accordingly, this study provides an evidence on Bambara groundnut diversity among the landraces that introduced from Africa (Nigeria) to Asia (Malaysia). All the modern applicable techniques may be applied for the betterment of this ongoing cultivated crops, but the dual approaches like conventional breeding linked with molecular breeding is highly successive over the solely use of one approach. However, traits improvement can be possible through direct selection with valuation of different genetic parameter analysis. The extent of selection approach exceedingly inspire by heritability and genetic gain estimation is the commanding tools for the enhancement of a certain traits^29^. Hence, the core intent of this study was to determine the inherent variation of Bamabara groundnut landraces using both qualitative and quantitative traits via valuation of characters association, variance component and different genetic parameters, resulting the identification of high yielding potentials from which pure lines will be developed for commercial cultivation.

## Materials and methods

### Experimental site

The research work has experimented under the Institute of Tropical Agriculture and Food Security (ITAFoS), University Agricultural Park, Universiti Putra Malaysia (UPM), Malaysia. Based on the Global Positioning System (GPS) the research location was 2°58ˊ54.0˝N latitude and 101°42ˊ53.8˝E longitude. The seeds of accessions were sown in open field conditions during the 2018–2019 cropping season. The soil PH is 6.6 to 7.5 with sandy loam to clay loam type (Dept. of land management, UPM). Fifteen accessions of Bambara groundnut were selected for this current research work, all representing the African accessions collected from the local market of Nigeria. Land races of Bambara groundnut used in this research was listed in Table 1. Randomly five plants were taken into consideration to evaluate genetic variability based on the agronomic traits^30^.

**Table 1:** The line-up of Bambara groundnut accessions with source of collection.

Population name & code	Locality	Sample size	Latitude (N)	Longitude (E)	Elevation (m)
Dai (G15), Hawayenzaki (G12)	Alkaleri, Buchi state	2	10.1558° N	10.207° E	616 m
Duna (G1), Maikai (G2), Cancaraki (G3), Jatau (G6), Katawa (G10), Giiwa (G9), Karu (G8)	Gombe state (central)	7	10.2791° N	11.1731° E	449 m
Roko (G4), Bidiyashi (G11)	Kwami, Gombe state	2	10.5085° N	11.2524° E	503 m
Ollel (G13), Bidillali (G5)	Akko, Gombe state	2	10.1186° N	11.0259° E	446 m
Exsokoto (G14), Maibergo (G7)	Sokoto state	2	13.0059° N	5.2476° E	450 m

### Experimental design

The experiment was conducted in a randomized complete block design (RCBD) with three replications. The experimental plot comprised of two rows measuring 1.6 m × 0.80 m. The distance between plant to the plant 30 cm, row to row 50 cm, plot to plot 1.5 m and the distance between replication was 2.0 m according to Unigwe et al*.*^30^. During the growing season the recommended intercultural practices like land preparation, land clearing, weeding, irrigation, and fertilizer were practiced. The recommended fertilizer rates (100% N = 45 kg N/ha, 100% P = 54 kg P_2_O_5_/ha, 100% K = 45 kg K_2_O/ha) and all portion of Phosphorus and Potassium were applied during field preparation hence, 70% N was applied at 5 weeks after sowing^31^.

### Parameters recorded for data analysis

Twenty-seven quantitative and 14 qualitative characters (Table 2) were considered during the morphological characterization. For comfort description, quantitative traits were categorized as (1) Phenological traits; (2) Growth and vegetative traits; (3) Yield traits. Following the Bambara groundnut description and descriptors states by IPGRI, IITA, BAMNET^32^ data were recorded from 5 randomly selected plants of each plot at several growth stages in the field and post-harvest data in the physiology lab.

**Table 2:** Twenty-seven quantitative and 14 qualitative traits measured according to IPGRI, IITA, BAMNET^32^.

Sl.No.	Quantitative trait	Code	Sl.No.	Qualitative trait	Code
**(1) Phenological trait (3)**			1	Growth habit	(GH)
1	Days to emergence	DTE (day)	2	Stem hairiness	(SH)
2	Days to fifty % flowering	D50%F (day)	3	First stem color	(FSC)
3	Days to maturity	DTM (day)	4	Terminal leaflet shape	(TLS)
**(2) Growth and vegetative trait (9)**			5	Terminal leaflet colour	(TLC)
4	Plant height	PH (cm)	6	Petiole pigmentation	(PP)
5	Number of branches per stem	NB	7	Shape of pods	(PS)
6	Number of stems per plant	NS	8	Colour of pods	(PC)
7	Number of petioles per plant	NP	9	Pods texture	(PT)
8	Number of leaves per plant	NL	10	Seeds shape	(SS)
9	Number of nodes per stem	NNS	11	Seeds colour	(SC)
10	Internode length	IL (cm)	12	Eyes color	(EC)
11	Biomass fresh weight per plant	BFW (g)	13	Testa pattern	(TP)
12	Biomass dry weight per plant	BDW (g)	14	Testa color with eye pattern around hilum	(TCEP)
**(3) Yield trait (15)**					
13	Total no. of pod per plant	TNP			
14	Mature pods number per plant	NMP			
15	Immature pods number per plant	NIP			
16	Fresh pod weight	FPW (g)			
17	Dry pod weight	DPW (g)			
18	Length of pod	PL (mm)			
19	Width of pod	PW (mm)			
20	Number of seed per	NSP			
21	Dry seed weight per plant	DSW (g)			
22	Length of seed	SL (mm)			
23	Width of seed	SW (mm)			
24	Hundred seed weight	HSW (g)			
25	Shelling percentage (%)	Shell%			
26	Harvest index	HI (%)			
27	Yield kg per hectare	Yld (kg/ha)			

### Statistical analysis

The SAS (statistical analysis software) version 9.3 was followed to test the significant differences using the analysis of variance (ANOVA) procedure at the level of LSD; P ≤ 0.05 and to compare among the mean of significant of traits. The correlations between the quantitative variables were determined using Pearson^33^ correlation coefficient formula. The genotypic and phenotypic variation were calculated as per following the formula given by Singh and Choudhary^34^. The coefficient variation of phenotypic (PCV) and genotypic (GCV): were estimated as per formula given by Khan et al*.*^23^ also relative differences was estimated using the formula (RD) = Relative difference between PCV and GCV. The estimated values of PCV and GCV were categorized by Robinson et al*.*^35^ and Khan et al*.*^23^, like as between 0 and 10% for low, 10–20% for intermediate and greater than (≥ 20%) for high. Broad sense heritability (${h}_{b}^{2}$) was estimated using the formula given by Falconer^36^ and Khan et al*.*^23^. In accordance with Johnson et al.^37^ and Khan et al.^23^, the heritability was categorized as between 0 and 30% for low, 30–60% for intermediate and greater than 60% as high. Genetic Advance (GA) (as a percentage of mean): was calculated with 5% selection intensity (K) following the method of^37^. Genetic advance is categorized as between 0 and 10% for low, 10% to 20% for intermediate and more (> 20%) than for high, following the formula given by Khan et al*.*^23^. K for constant also indicates the intensity of selection. According to Adewale et al*.*^38^ the rate is 2.06 at the point when the K is at 5%. Genetic Gain (%) = Estimated as genetic advance (GA) × 100; it is also categorized^37,]^^39^ as between (0 to 10%) for low, (10 to 20%) for intermediate and (≥ 20%) for high GA. Based on the Euclidian Distance Method also Dices’s and Jaccard’s similarity of coefficient data was analysed for investigation of genetic diversity. In addition to this, based on the Unweighted Pair Group Method using Arithmetic Average (UPGMA) and following the algorithm & sequential, agglomerative, hierarchic, and non-overlapping (SAHN) method the genetic inter-relationship (showing dendrogram) among the Bambara groundnut were estimated. For this analysis NTSYS version 2.1 (Numerical Taxonomy Multivariate Analysis System), Exeter Software, Setauket, NY, USA software^40^ were used. Using similar software, the principal component analysis (PCA) was done to produce two dimensional (2D) plots though, multivariate statistical packages (MVSP) was used for PCA biplot loading. However, the Shannon diversity index is a synonym for the Shannon equitability index and evenness was calculated using the formula given by Shannon^41^ and Hennink and Zeven^42^. For germplasm selection, rank summation index (RSI) was estimated using Onwubiko et al*.*^43^; Mulumba and Mock^44^ reported formula. For correlation networking, pattern search plot we used MDP tools while for heatmap study we used ClustVis bio tools.

## Results

### Assessment of qualitative variation

**Figure 1:**
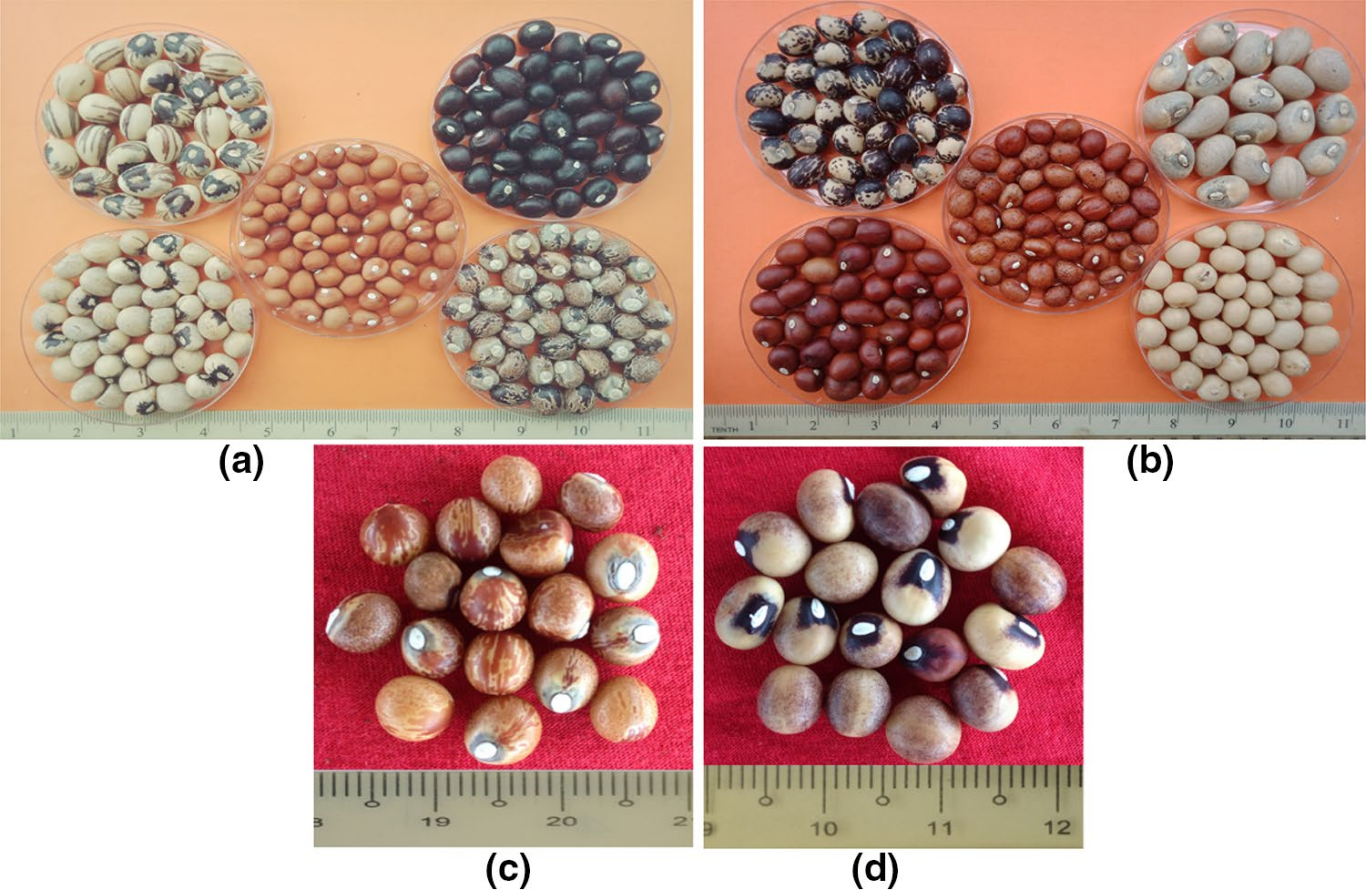
Some qualitative features of Bambara groundnut seeds: large and irregular eye pattern [two thick line join together forming an almost triangular shape **a** (top left), **a** (down left), & **d**]; thin and sharp circle around the eye and very light strip with creamy seedcoat [**b** (top right)]; No eye pattern [**a** (down right), **a** (top right), **a** (centre), **b** (top left, down left, down right, centre) & **c**]; Black stripped on one side of hilum [**a** (top left)]; Marbled with reddish seed coat [**b** (centre)]; Few rhomboid spots on one side of hilum (**c**); many rhomboid spots almost covering the entire hilum of both side [**a** (down right), **b** (top left)]; brownish seed coat [**a** (centre)]; cream seed coat [**b** (down right)]; dark reddish seed coat [**b** (down left)]; black seed coat [**a** (top right)]; cream with blackish seed coat (**d**).

**Figure 2:**
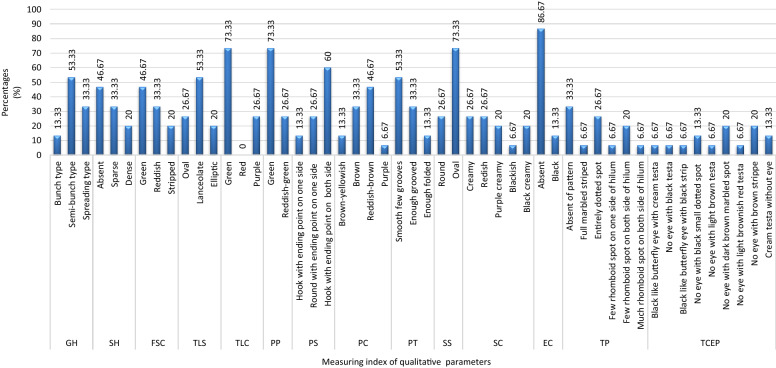
Graphical display of qualitative traits’ frequency distribution of Bambara groundnut accessions. *GH* growth habits, *SH*stem hairiness, *FSC *color of first stem, *TLS* shape of terminal leaflet, *TLC* terminal leaf color, *PP* petiole pigmentation, *PS* pod shape, *PC* pod color, *PT* pod texture, *SS* seed shape; *SC* seed color; *EC* eye color, *TP* Testa pattern, *TCEP* Testa color with eye pattern round hilum.

The frequency of distribution of some qualitative variables are summarized in Fig. 1 and Fig. 2. After two weeks later, we observed 46.67% of the accessions had greenish stems, 20% had stripped stems and 33.33% were reddish stems.

The terminal leaflets had two different colours: 73.33% accession had a greenish leaflet while the purple was 26.67% accessions. The 53.33% of the total accessions had terminal leaflets shaped like lanceolate whereas 26.67% had oval and 20% had elliptic in shape. Among the 15 characterized accession, three growth habits were found: bunch type accessions (13.33%), semi bunch type accessions (53.33%) and the spreading type was (33.33%). Among the landraces, 33.33% had sparse hair on their stems and 20% had dense hair while 46.67% did not have any. Most of the landraces had reddish-brown (46.67%) and brown (33.33%) colour pods some had yellowish-brown (13.33%), and purple (6.67%) colour pods. Maximum accessions were found oval (73.33%) seeds shape and few had round (26.67%). Seed colour had cream and red (26.67%), black cream and cream purple (20.00%), only 6.67% had black colour. 13.33% of landraces had black eye color and 86.67% had no eye colour.

### Estimation of quantitative parameters

#### Morphological diversity

For crop upgrading plant breeders considered yield and its related traits as a controlling parameter. In current research, to select the best performing accessions for next breeding program a total number of 27 numerical traits of 15 Bambara groundnut landraces were analysed. The analysis of variance revealed the significant variation, mean, standard error of mean (SE.m), standard deviation (St.Dev), and coefficient of variation (CV%) displayed in Table 3. Except the traits (fifty percent flowering day, seed length, seed width) all other quantitative traits showed highly significant (P ≤ 0.01) difference. No significant difference was found for the trait plant height (24.36 cm ± 0.39). Within replication no significant variation was observed. The highest and lowest values for the respective traits across all plants were presented in Table 3. We found 16 out of the 27 quantitative traits had coefficient of variation (CV) ≥ 20% which ranged from 5.96% (Shelling%) to 58.55% (dry seed weight per plant (g). The average days to maturity was found (131.56 ± 1.18) days which is statistically significant (p ≤ 0.01). The highest value of standard deviation (SD) was found for yield kg/ha (650.98) with standard error (SEm ± 97.04) while the lowest was for internode length (SD = 0.49; SEm ± 0.07) (Table 3). Standard error (SE) is the indication of consistency of the average values, lowest SE values indicate the sample mean is more precise reflection of the real population mean.

**Table 3:** Summary of the signification variation revealed by analysis of variance (ANOVA).

Trait		Replications (df = 2)	Genotypes (df = 14)	Mean ± SEm	Max	Min	St. dev	CV (%)
**Phenological trait**								
DTE		0.42	7.85**	7.96 ± 0.27	13.00	5.00	1.81	22.73
D50%F		6.20	41.24*	39.27 ± 0.74	49.00	31.00	4.98	12.68
DTM		6.16	172.46**	131.56 ± 1.18	144.00	118.00	7.93	6.03
**Vegetative trait**								
PH		7.69	9.18 ns	24.36 ± 0.39	29.46	16.21	2.60	10.69
NB		0.82	295.08**	34.82 ± 1.47	59.00	12.00	9.86	28.32
NS		6.02	21.68**	13.36 ± 0.51	25.00	7.00	3.39	25.40
NP		451.47	17,849**	298 ± 11.68	410.00	150.00	78.34	26.29
NL		4063.2	160,641**	894 ± 35.07	1230.00	450.00	235.01	26.29
NNS		5.40	6.91**	11.6 ± 0.28	16.00	8.00	1.92	16.58
IL		0.25	0.46**	3.15 ± 0.07	4.60	2.40	0.49	15.66
BFW		473.98	57,774.96**	316.85 ± 20.38	621.00	92.70	136.72	43.15
BDW		1396.43	18,453.82**	141.012 ± 11.88	373.64	43.41	79.74	56.55
**Yield trait**								
TNP		25.27	488.09**	67.67 ± 1.97	96.00	48.00	12.92	19.10
NMP		7.22	403.40**	51.69 ± 1.76	78.00	33.00	11.79	22.80
NIP		5.49	45.83**	15.98 ± 0.66	26.00	8.00	4.45	27.85
FPW		66.84	49,298.44**	281.46 ± 18.70	589.00	145.50	125.44	44.57
DPW		328.36	18,889.72**	141.64 ± 11.65	375.13	64.72	78.12	55.15
PL		1.32	125.31**	27.48 ± 0.96	44.47	17.96	6.45	23.49
PW		0.86	7.82**	13.17 ± 0.28	18.59	9.42	1.88	14.29
NSP		33.01	488.09**	66.17 ± 1.93	95.00	46.00	12.94	19.55
DSW		30.27	11,220.87**	102.47 ± 8.94	277.71	46.88	59.99	58.55
SL		0.83	7.76*	13.27 ± 0.32	20.45	9.33	2.13	16.06
SW		0.22	2.39*	9.07 ± 0.18	11.57	6.42	1.21	13.34
HSW		1.09	11.49**	228.02 ± 7.71	389.32	160.98	51.73	22.69
Shel%		6.94	39.71**	71.79 ± 0.64	79.97	59.29	4.28	5.96
HI		57.44	433.91**	50.46 ± 1.87	83.88	33.82	12.54	24.85
Yld		22,803.08	1,311,786.57**	1180.34 ± 97.04	3126.00	539.33	650.98	55.15

*df* degree of freedom; *ns* non-significant; *SEm* standard error of the mean; *St. Dev* standard deviation; *Max* maximum; *Min* minimum; P ≤ 0.05 = significant (*); P ≤ 0.01 = highly significant (**), *CV* coefficient of variation for error, *DTE (day)* days to emergence, *D50%F (day)* days to 50% flowering, DTM (day) days to maturity, *PH* plant height (cm), *NB* number of branches per plant, *NS* number of stems per plant, *NP* number of petioles per plant, *NL* number of leaves per plant, *NNS* no. of nodes per stem, *IL* (cm) inter nodes length, *BFW (g)* biomass fresh weight per plant, *BDW (g)* biomass dry weight per plant, *TNP* total no. of pods per plant, *NMP* number of mature pods per plant, *NIP* number of immature pods per plant, *FPW (g)* fresh pods weight, *DPW (g)* dry pods weight, *PL (mm)* pod length, *PW (mm)* PW, *NSP* number of seeds per plant, *DSW (g)* dry seed weight per plant, *SL (mm)* seed length, *SW (mm)* seed width, *HSW (g)* hundred seed weight, *Shel%* shelling percent, *HI *(%) harvest index ,*Yld *(kg ha^−1^) yield .

**Figure 3:**
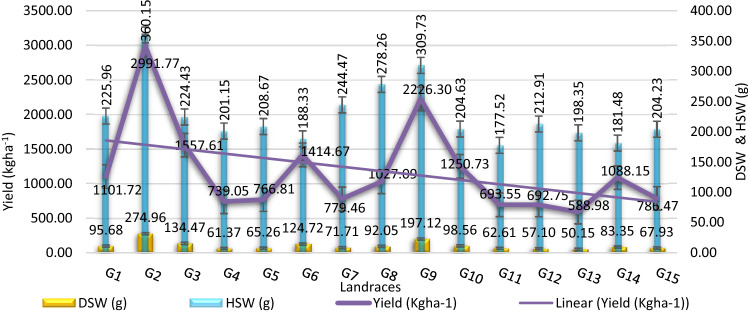
Graphical relationship of dry seed weight (DSW) and hundred seed weight (HSW) with yield (kg ha^−1^) for Bambara groundnut landraces.

The maximum and minimum values for overall accessions and mean comparison with least significant difference (LSD = 0.05) were shown in the Tables 4 and 5. Fig. 3 showing the graphical relationship of DSW(g) and HSW(g) with yield (kg ha^−1^). The days to 50% flowering varied from 31 to 49 days after sowing (DAS) while 66.67% of the accessions gave 50% flower before 40 days after sowing. Most of the landraces (80%) took more than 120 days to maturity which varied from 122 to 141 DAS. The genotype G1 marked as short duration line with a total day to maturity of 119 DAS (Table 4). The genotype G2 recorded highest values for the traits like—BFW (614.67 g); BDW(361.57 g) (Table 4); TNP (93.67); NMP (74.33); FPW (568.56 g); DPW (359.01 g); NSP (92.17); DSW (274.96 g); SW(10.74 mm); HSW(360.15 g); Sell% (76.68%) and yield (2991.77 kg ha^−1^) (Table 5) while the accession G13 had lowest values. The next maximum yield (kg ha^−1^) was recorded for the genotype G9 (2226.30 kg ha^−1^) followed by G3 (1557.61 kg ha^−1^), G6 (1414.67 kg ha^−1^) and G10 (1250.73 kg ha^−1^) (Tables 4 and 5).

**Table 4:** The mean and mean comparison of 3 phenological and 9 vegetative traits of 15 Bambara groundnut accessions.

Genotype	DTE	D50%F	DTM	PH	NB	NS	NP	NL	NNS	IL	BFW	BDW
**G1**	5.33d	39.33bcd	119.67f	25.56a-d	44.67b	11cde	349.33b-e	1048b-e	13ab	3.53ab	364.24c	150.57d
G2	7.33bc	37.33bcd	121f	23.87a-e	34de	16.67a	279.33g	838g	12abcd	2.97bcd	614.67a	361.57a
G3	7.33bc	35d	132.33bcd	26.72ab	32ef	11.67b-e	179.33i	538i	12.33abc	3bcd	296.8de	140.39d
G4	6.67cd	43.67abc	139.67a	22.31cde	13h	9.67de	304.33fg	913fg	9.33ef	2.7d	188.04hg	62.39f
G5	8.33bc	38.67bcd	138.33a	24.08a-e	35de	12.67a-d	316.67efg	950efg	10.33c-f	3.83a	329.83cd	146.77d
G6	6.67cd	44.33ab	129d	25.87abc	40.33c	15abc	369.67abc	1109abc	13ab	3.83a	485.57b	218.55b
G7	11a	37.33bcd	138.33a	23.11b-e	36.67cd	16ab	328.67c-f	986c-f	11b-f	2.93bcd	344.84c	160.81cd
G8	9b	34.33d	122.33ef	25.23a-e	56.67a	16.67a	165i	495i	12.33abc	3.3a-d	511b	197.68cb
G9	8bc	37.33bcd	121.67f	27.14a	45b	16.33a	361.67a-d	1085a-d	11.67b-e	3.2bcd	155.98h	62.84f
G10	7.67bc	37.67bcd	136abc	23.65a-e	31.33ef	12.67a-d	226.66 h	680 h	12.67abc	3.4abc	275.66e	78.56f
G11	7cd	37cd	130.33 cd	23.8a-e	35de	16ab	310efg	930efg	11b-f	2.8 cd	195.55g	87.19ef
G12	7.33bc	47.67a	138ab	21.92de	34.33de	13.33a-d	177.33i	532i	9.67def	2.73d	359.38c	122.04de
G13	8bc	42.33abc	128de	21.57e	23.67g	12.33a-e	402a	1206a	9f	2.77d	109.97i	50.22f
G14	8bc	40bcd	141.33a	26.45ab	31.67ef	8e	381.67ab	1145ab	12.33abc	3.5ab	286.51e	141.03d
G15	11.67a	37cd	137.33ab	24.13a-e	29f	12.33a-e	318.33d-g	955d-g	14.33a	2.77d	234.68	134.63d
Mean	7.96	39.27	131.56	24.36	34.82	13.36	298.00	894.00	11.60	3.15	316.85	141.02
LSD	1.82	7.07	5.83	ns	3.82	4.36	43.83	131.50	2.35	0.60	35.65	43.15
Min	5.33	34.33	119.67	21.57	13.00	8.00	165.00	495.00	9.00	2.70	109.97	50.22
Max	11.67	47.67	141.33	27.14	56.67	16.67	402.00	1206.00	14.33	3.83	614.67	361.57

*Max* maximum, *Min* minimum, *C.V. *coefficient of variation, *LSD* least significant difference at 5% level, *DTE (day)* days to emergence, *D50%F (day)* days to 50% flowering, *DTM (days)* days to maturity, *PH* plant height (cm), *NB* number of branches per plant, *NS* number of stems per plant, *NP* number of petioles per plant, *NL* number of leaves per plant, *NNS* no. of nodes per stem, *IL* (cm) inter nodes length, *BFW (g)* biomass fresh weight per plant, *BDW (g)* biomass dry weight per plant .

**Table 5:** The mean and mean comparison of 15 yield related traits of 15 Bambara groundnut accessions.

Genotype	TNP	NMP	NIP	FPW	DPW	PL	PW	NSP	DSW	SL	SW	HSW	Shel%	HI	Yld
**G1**	71.67de	53.33c	18.33abc	188.59g	132.21de	24.79d	12.24cd	70.17de	95.68d	12.91bc	9.76abc	225.96de	72.35a-d	46.92def	1101.72de
G2	93.67a	74.33a	19.33ab	568.55a	359.01a	38.68b	15.48ab	92.17a	274.96a	16.29a	10.74a	360.15a	76.68a	49.82cd	2991.77a
G3	78.67cb	57c	21.67a	325.06d	186.91c	25.34d	13.19c	77.17cb	134.47c	13.71abc	9.19abc	224.43def	71.93a-d	57.13cb	1557.61c
G4	50.67j	40.33efg	10.33e	186.22g	88.68 fg	20.95ef	12.25 cd	49.17j	61.36 fg	11.48c	8.90bc	201.15e-i	69.11cde	58.69b	739.05 fg
G5	59.67fgh	41efg	18.67ab	157.17i	92.02f	24.51d	12.75 cd	58.17fgh	65.26 fg	12.43bc	8.64bc	208.67efg	70.96b-e	38.56fg	766.81f
G6	65.33ef	54.67c	10.67e	332.97d	169.76c	24.15d	14.05bc	63.83ef	124.72c	13.75abc	9.18abc	188.33ghi	73.54abc	44.34d-g	1414.67c
G7	57.67ghi	45.33de	12.33de	180.95gh	93.53f	30.27c	13.51bc	56.17ghi	71.71f	12.95bc	9.5abc	244.46d	76.67a	36.86g	779.47
G8	81.33b	67.67ab	13.67cde	391.64c	123.25e	31.56c	14.09bc	79.83b	92.05de	13.75abc	9.32abc	278.25c	74.66ab	39.07fg	1027.09e
G9	83.67b	66.67b	17a-d	469.11b	267.16b	32.39c	16.63a	82.17b	197.12b	16.62a	9.94ab	309.73b	73.84abc	81.10a	2226.3b
G10	72cde	56c	16bcd	281.08e	150.09d	42.70a	13.27c	70.5cde	98.56d	12.71bc	8.19cd	204.63e-h	66.49ef	65.60b	1250.73d
G11	60fgh	51cd	9e	174.75gf	83.22fg	19.56f	9.89e	58.5fgh	62.61fg	11.43c	9.09abc	177.52i	75.22ab	48.87cde	693.54fg
G12	61.67gf	42.66ef	19ab	167.14hi	83.12fg	24.69d	13.22c	60.17gf	57.09gh	13.21bc	8.98bc	212.91efg	68.62def	40.56efg	692.74fg
G13	51.67ij	35g	16.67bcd	155.5i	70.67g	24.11d	11.03de	50.17ij	50.15 h	11.03c	6.65d	198.35f-i	71.00b-e	58.58b	588.97g
G14	54.67hij	38fg	16.67bcd	388.44c	130.56e	23.31de	12.64 cd	53.17hij	83.35e	12.32bc	9.03abc	181.48hi	63.83f	48.58de	1088.15e
G15	72.67 cd	52.33c	20.33ab	254.63f	94.38f	25.14d	13.26c	71.17cd	67.93fg	14.49ab	8.88bc	204.23e-i	71.99a-d	42.22d-g	786.47f
Mean	67.67	51.69	15.98	281.46	141.64	27.48	13.17	66.17	102.47	13.27	9.07	228.02	71.80	50.46	1180.34
LSD	6.82	6.70	4.67	14.37	18.41	2.75	2.11	6.82	10.98	2.99	1.74	26.75	4.86	8.53	153.39
Min	50.67	35.00	9.00	155.50	70.68	19.56	9.90	49.17	50.15	11.03	6.65	177.52	63.83	36.86	588.98
Max	93.67	74.33	21.67	568.56	359.01	42.70	16.63	92.17	274.96	16.62	10.74	360.15	76.68	81.10	2991.77

*Max* maximum, *Min* minimum, *C.V.* coefficient of variation, *LSD* least significant difference at 5% level, *TNP* total no. of pods per plant, *NMP* number of mature pods per plant, *NIP* number of Immature pods per plant, *FPW (g)* fresh pods weight, *DPW(g)* dry pods weight, *PL(mm)* pod length, *PW (mm)* PW, *NSP* number of seeds per plant, DSW(g) dry seed weight per plant, *SL(mm)* seed length, *SW (mm)* seed width, *HSW(g)* hundred seed weight, *Shel%* shelling percent, *HI (%)* harvest index, *Yld (kg/ha)* yield.

### Analysis of correlation matrix

**Figure 4:**
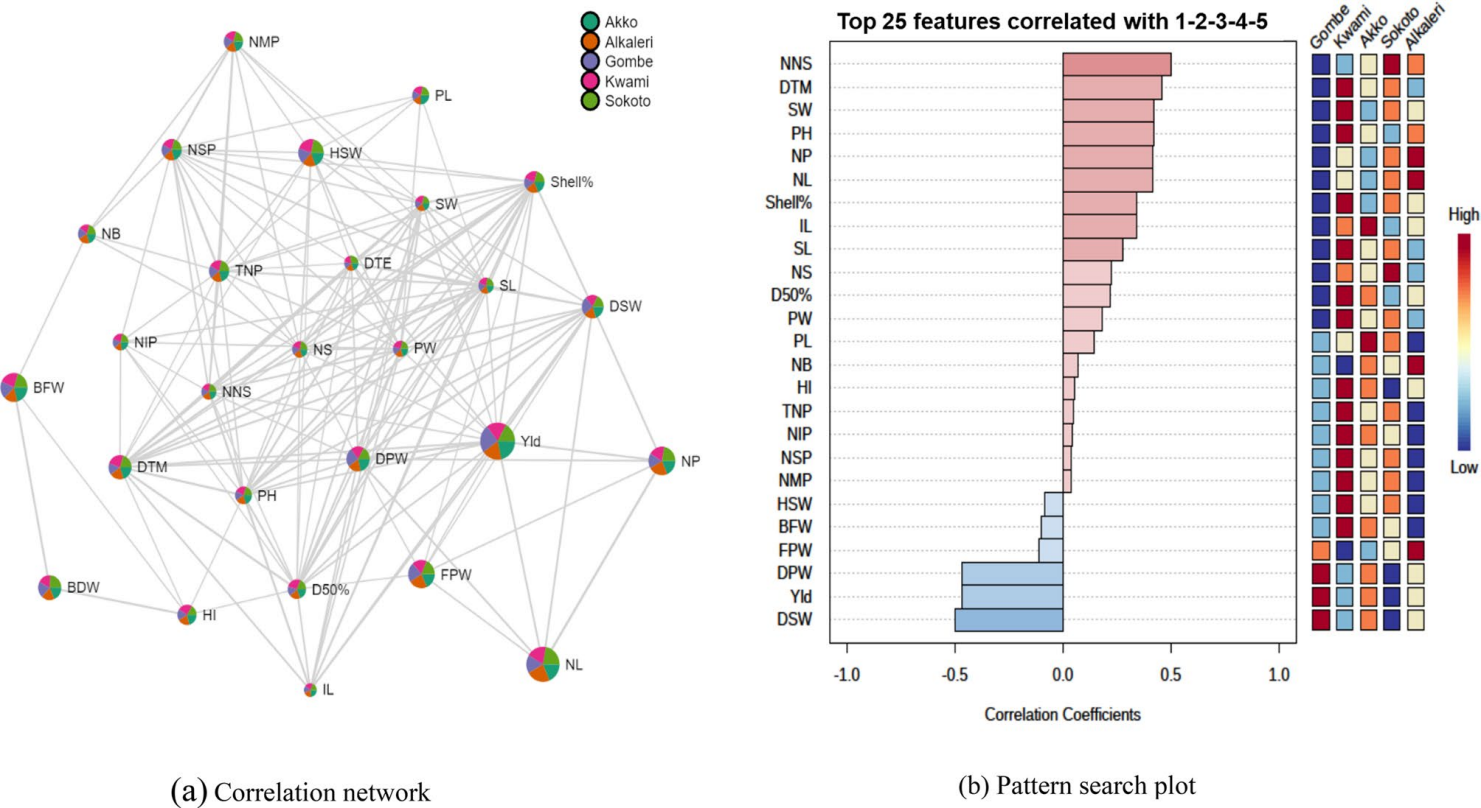
**(a)** Correlation network illustrates the relationship of 27 quantitative descriptors with five geographic population. Each node represents 5 geographic population of BG with five defined color and each edge represent the correlation between the two traits. **(b)** Pattern search plot insight into the correlation abundance of top 25 traits of respective five (1 = Gombe; 2 = Kwami; 3 = Akko; 4 = Sokoto; 5 = Alkaleri) Bambara ground nut population. Days to emergence = DTE (day), days to 50% flowering = D50%F (day), days to maturity = DTM (day), plant height (cm) = PH, number of branches per plant = NB, number of stems per plant = NS, number of petioles per plant = NP, number of leaves per plant = NL, no. of nodes per stem = NNS, inter nodes length = IL (cm), biomass fresh weight per plant = BFW(g), biomass dry weight per plant = BDW(g), total no. of pods per plant = TNP, number of mature pods per plant = NMP, number of Immature pods per plant = NIP, fresh pods weight = FPW(g), dry pods weight = DPW(g), pod length = PL(mm), PW = PW (mm), number of seeds per plant = NSP, dry seed weight per plant = DSW(g), seed length = SL(mm), seed width = SW (mm), hundred seed weight = HSW(g), shelling percent = Shel%, harvest index = HI (%) and yield = Yld (kg ha^−1^).

The phenotypic correlation among the 27 numerical traits of fifteen Bambara groundnut accessions is given in Table 6. Days to 50% flowering showed negative and intermediate (0.25 ≤ r < 0.75) significant association with NB ( r = −0.29; P = 0.04), NNS(r = 0.33; P = 0.02), TNP (r = −0.38; P = 0.00), NMP (r = −0.35; P = 0.01) and NSP (r = −0.38; P = 0.00). A positive and highly strong (0.75 ≤ r < 1) significant association was found for the traits like NMP (r = 0.93; P ≤ 0.00), DPW (r = 0.76; P ≤ 0.00), NSP (r = 0.99; P ≤ 0.00), DSW (r = 0.77; P ≤ 0.00) and yield (r = 0.76; P ≤ 0.00) with the total number of pods. The trait yield (kg ha^−1^) revealed positive and perfect (r = 1.00) highly significant association with DPW (r = 1.00; P ≤ 0.00) while positive and highly strong ( 0.75 ≤ r < 1) significant association was found with DSW (r = 0.99; P ≤ 0.00), NSP (r = 0.76; P ≤ 0.00) and HSW (r = 0.76; P ≤ 0.00) per plant (Table 6). Correlation networking and pattern search plot are more visual illustration of correlation matrix. In correlation network (Fig. 4a) each node represents a variable with its colour based on the defined geographical population (Gombe-7 accessions, Kwami- 2 accessions, Akko- 2 accessions, Alkaleri- 2 accessions, and Sokoto- 2 accessions) group and its size is based on number of correlations to that variable. The traits yield, DPW, FPW, HSW, NP, and NL showed larger node size then other traits. Two variables are connected by an edge if the correlation between the two variables meet the p-value (0.05) and thresholds. The edge size also reflects the magnitude of the correlation. Helps in identifying biologically meaningful relationship or associations between group or features. On other hand, pattern search (Fig. 4b) plot showed the (max) top 25 features correlated with the sample of interest. The features are ranked by their correlation, and the blue bars represent −ve correlations while red bar indicates + ve correlations. The deeper the color (darker blue or red), the stronger the correlation. To the right mini heatmap showing whether the abundance of that features is higher (red) or lower (blue) in each population group.

**Table 6:** Pearson’s Correlation matrix (r) for 27 quantitative traits of Bambara groundnut accessions.

Trait	DTE	D50F	DTM	PH	NB	NS	NP	NL	NNS	IL	BFW	BDW			
DTE	1	− 0.178	0.258	− 0.055	0.017	0.229	− 0.017	− 0.017	0.086	− 0.224	− 0.076	− 0.008			
D50%F		1	0.182	− 0.206	− 0.293*	− 0.057	0.125	0.125	− 0.333*	0.042	− 0.077	− 0.2			
DTM			1	− 0.233	− 0.554**	− 0.386**	− 0.065	− 0.065	− 0.152	− 0.124	− 0.299*	− 0.287*			
PH				1	0.330*	0.129	0.072	0.072	0.310*	0.352*	0.114	0.135			
NB					1	0.423*	− 0.187	− 0.187	0.365*	0.376*	0.495**	0.333*			
NS						1	− 0.13	− 0.13	− 0.047	0.095	0.286	0.249			
NP							1	1.00**	− 0.007	0.21	− 0.347*	− 0.16			
NL								1	− 0.007	0.21	− 0.347*	− 0.16			
NNS									1	0.293*	0.274	0.341*			
IL										1	0.281	0.161			
BFW											1	0.903**			
BDW												1			

Trait	TNP	NMP	NIP	FPW	DPW	PL	PW	NSP	DSW	SL	SW	HSW	Shel%	HI	Yld
DTE	0.052	0.013	0.116	− 0.016	− 0.204	0.121	0.139	0.051	− 0.172	0.111	− 0.099	0.087	0.231	− 0.25	− 0.204
D50%F	− 0.387**	− 0.357*	− 0.177	− 0.265	− 0.205	− 0.283	− 0.056	− 0.389**	− 0.212	− 0.231	− 0.109	− 0.245	− 0.232	0.049	− 0.205
DTM	− 0.629**	− 0.669**	− 0.055	− 0.445**	− 0.531**	− 0.262	− 0.268	− 0.629**	− 0.563**	− 0.301*	− 0.203	− 0.541**	− 0.480**	− 0.274	− 0.531**
PH	0.331*	0.344*	0.049	0.391**	0.304*	0.068	0.344*	0.337*	0.282	0.330*	0.382*	0.141	− 0.005	0.163	0.304*
NB	0.514**	0.558**	0.015	0.354*	0.247	0.273	0.311*	0.513**	0.262	0.328*	0.340*	0.393**	0.349*	− 0.172	0.247
NS	0.390**	0.481**	− 0.143	0.245	0.330*	0.331*	0.403**	0.387**	0.362*	0.214	0.352*	0.465**	0.501**	0.026	0.330*
NP	− 0.372*	− 0.341*	− 0.176	− 0.088	− 0.034	− 0.278	− 0.142	− 0.369*	− 0.036	− 0.118	− 0.082	− 0.195	− 0.045	0.166	− 0.034
NL	− 0.372*	− 0.341*	− 0.176	− 0.088	− 0.034	− 0.278	− 0.142	− 0.369*	− 0.036	− 0.118	− 0.082	− 0.195	− 0.045	0.166	− 0.034
NNS	0.428*	0.429*	0.105	0.343*	0.241	0.227	0.141	0.431*	0.233	0.388**	0.291*	0.027	0.08	− 0.133	0.241
IL	0.046	0.063	− 0.034	0.142	0.139	0.105	0.211	0.053	0.114	− 0.058	0.125	− 0.08	− 0.113	− 0.028	0.139
BFW	0.489**	0.517**	0.053	0.481**	0.462**	0.375*	0.354*	0.489**	0.486**	0.306*	0.421**	0.477**	0.320*	− 0.497**	0.462**
BDW	0.514**	0.525**	0.102	0.559**	0.570**	0.326*	0.371*	0.515**	0.600**	0.432**	0.467**	0.533**	0.386**	− 0.498**	0.570**
TNP	1	0.939**	0.417**	0.741**	0.763**	0.615**	0.582**	0.999**	0.779**	0.649**	0.442**	0.733**	0.382*	0.199	0.763**
NMP		1	0.08	0.746**	0.744**	0.594**	0.583**	0.939**	0.764**	0.615**	0.464**	0.715**	0.455**	0.19	0.744**
NIP			1	0.177	0.246	0.213	0.145	0.417**	0.238	0.255	0.055	0.236	− 0.095	0.076	0.246
FPW				1	0.877**	0.546**	0.641**	0.741**	0.869**	0.580**	0.416**	0.700**	0.165	0.306*	0.877**
DPW					1	0.589**	0.647**	0.761**	0.993**	0.583**	0.486**	0.768**	0.238	0.404**	1.000**
PL						1	0.551**	0.615**	0.572**	0.403**	0.235	0.604**	0.062	0.279	0.589**
PW							1	0.578**	0.631**	0.603**	0.429**	0.628**	0.066	0.217	0.647**
NSP								1	0.777**	0.651**	0.443**	0.730**	0.386**	0.197	0.761**
DSW									1	0.589**	0.483**	0.800**	0.338*	0.366*	0.993**
SL										1	0.665**	0.587**	0.253	0.084	0.583**
SW											1	0.490**	0.207	− 0.07	0.486**
HSW												1	0.483**	0.181	0.768**
Shel%													1	− 0.183	0.238
HI														1	0.404**
Yld															1

“**” correlation is significant at the 0.01 level; “*” correlation is significant at the 0.05 level, *DTE (day)* days to emergence, *D50%F (day)* days to 50% flowering, *DTM (day)* days to maturity, *PH* plant height (cm), *NB* number of branches per plant, *NS* number of stems per plant, *NP* number of petioles per plant, *NL* number of leaves per plant, *NNS* no. of nodes per stem, *IL* (cm) inter nodes length, *BFW(g)* biomass fresh weight per plant, *BDW(g)* biomass dry weight per plant, *TNP* total no. of pods per plant, *NMP* number of mature pods per plant,* NIP* number of immature pods per plant, *FPW(g)* fresh pods weight, *DPW(g)* dry pods weight, *PL (mm)* pod length, *PW (mm)* PW, *NSP* number of seeds per plant, *DSW(g)* dry seed weight per plant, *SL(mm)* seed length, *SW (mm)* seed width, *HSW(g)* hundred seed weight, *Shel%* shelling percent, *HI (%)* harvest index, *Yld* (kg ha^−1^) yield.

### Abundance (richness) analysis for traits

**Figure 5:**
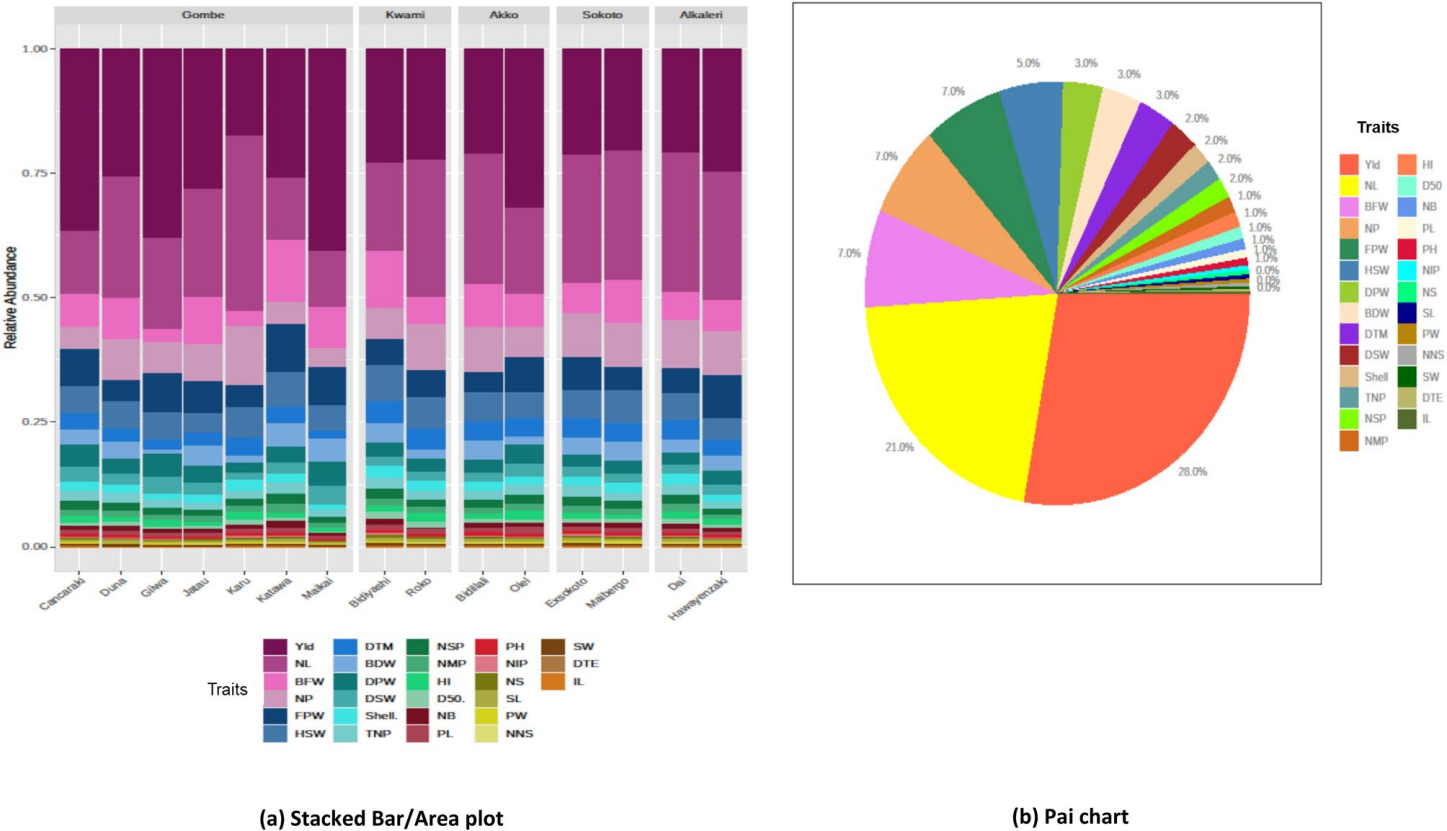
Relative abundance sketching: **(a)** stacked bar/area plot showing richness of traits in each genotype and **(b)** Pai chart showing the richness percentages with a unique color code for each trait.

Abundance sketching (Stacked Bar) provides overall as well as comparative abundance profile across landraces at different variable levels (Fig. 5a). Variation present in data of multiple variable level can be visualized for all individual genotypes wise. It also summarized and compared the abundance of different variables based on the multiple data. The pie chart provides an exact clue for all variables at multiple variation level present in data. Pie chart helps in visualizing the degree of diversity or abundance of variables compositions for different BG samples. Provides exact composition of each group through direct quantitative comparison of abundances in percentages (Fig. 5b). In the “Stacked bar or Area plot” each bar represents an individual genotype under the respective location (at the top of area olot). Among the traits, yield, NL, and BFW possess the higher area with showing different colour for each genotype however, according to pic chart these traits also occupied 28%, 21% and 7%, respectively.

### Analysis of genetic components

#### Variance and covariance, heritability in broad sense, relative differences, and genetic advances

The output of genetic components analysis was compiled in Table 7. Apparently, the phenotypic variance (${\sigma }_{p}^{2}$) is higher than the genotypic variance (${\sigma }_{g}^{2}$) regarding all the traits evaluated. The trait grain yield kg ha^−1^ reported higher genotypic (434,458.5) and phenotypic (442,869.6) variance while the lower genotypic (0.11) and phenotypic (0.24) value was recorded for the trait internode length. The traits such as TNP (PCV 19.48% & GCV 18.53%), PW (PCV 14.55% & GCV 10.95%), NSP (PCV 19.92% and GCV 18.95%), SL (PCV 16.37% & GCV 9.30%), SW (PCV 13.61% & GCV 7.29%) and shell% (PCV 6.05% & GCV 4.50%) showed below 20% of phenotypic coefficient of variation (PCV) and genotypic coefficient of variation (GCV). For the improvement of this crop further selection could be done considering the traits having GCV ≥ 20% (BFW, BDW, FPW, DPW, DSW and grain yield) which indicated high degree of variability among these traits although the variation is due to the effect of additive genes. Due to the lower GCV values (≤ 10%) the vegetative traits (PH, D50%F, and DTM) and yield traits (SL, SW, and Shel%) indicated the limited chance of selection based on respective traits due to the effect of environment on their phenotypic expression.

**Table 7:** Variance components, relative difference, heritability and genetic advance of 27 quantitative traits.

Trait	Mean	${\sigma }_{e}^{2}$	${\sigma }_{g}^{2}$	${\sigma }_{p}^{2}$	PCV (%)	GCV (%)	RD (%)	(${h}_{b}^{2}$) %	GA (%)
**Phenological trait**									
DTE	7.96 ± 0.27	1.18	2.22	3.41	23.20	18.74	19.23	65.24	31.18
D50%F	39.27 ± 0.74	17.89	7.79	25.68	12.90	7.11	44.93	30.32	8.06
DTM	131.56 ± 1.18	12.16	53.43	65.59	6.16	5.56	9.74	81.47	10.33
**Vegetative trait**									
PH	24.36 ± 0.39	5.52	1.22	6.74	10.66	4.53	57.46	18.09	3.97
NB	34.82 ± 1.47	5.20	96.63	101.83	28.98	28.23	2.59	94.89	56.65
NS	13.36 ± 0.51	6.81	4.96	11.77	25.69	16.68	35.08	42.15	22.30
NP	298 ± 11.68	686.82	5720.70	6407.52	26.86	25.38	5.51	89.28	49.40
NL	894 ± 35.07	6181.40	51,486.50	57,667.90	26.86	25.38	5.51	89.28	49.40
NNS	11.6 ± 0.28	1.97	1.65	3.62	16.40	11.07	32.53	45.53	15.38
IL	3.15 ± 0.07	0.13	0.11	0.24	15.65	10.65	31.95	46.31	14.93
BFW	316.85 ± 20.38	454.27	19,106.90	19,561.17	44.14	43.63	1.17	97.68	88.82
BDW	141.012 ± 11.88	665.71	5929.40	6595.11	57.59	54.61	5.18	89.91	106.66
**Yield trait**									
TNP	67.67 ± 1.97	16.65	157.15	173.80	19.48	18.53	4.91	90.42	36.29
NMP	51.69 ± 1.76	16.06	129.12	145.17	23.31	21.98	5.69	88.94	42.71
NIP	15.98 ± 0.66	7.80	12.68	20.48	28.32	22.28	21.31	61.91	36.12
FPW	281.46 ± 18.70	73.85	16,408.20	16,482.05	45.61	45.51	0.22	99.55	93.54
DPW	141.64 ± 11.65	121.12	6256.20	6377.32	56.38	55.84	0.95	98.10	113.94
PL	27.48 ± 0.96	2.70	40.87	43.57	24.02	23.26	3.15	93.79	46.41
PW	13.17 ± 0.28	1.59	2.08	3.67	14.55	10.95	24.74	56.64	16.97
NSP	66.17 ± 1.93	16.65	157.15	173.80	19.92	18.95	4.91	90.42	37.11
DSW	102.47 ± 8.94	43.08	3725.90	3768.98	59.91	59.57	0.57	98.86	122.01
SL	13.27 ± 0.32	3.20	1.52	4.72	16.37	9.30	43.21	32.25	10.88
SW	9.07 ± 0.18	1.09	0.44	1.52	13.61	7.29	46.42	28.71	8.05
HSW	228.02 ± 7.71	255.82	2525.90	2781.72	23.13	22.04	4.71	90.80	43.27
Shel%	71.79 ± 0.64	8.45	10.42	18.87	6.05	4.50	25.69	55.22	6.88
HI	50.46 ± 1.87	26.04	135.96	162.00	25.22	23.11	8.39	83.93	43.61
Yld	1180.34 ± 97.04	8411.10	434,458.50	442,869.60	56.38	55.84	0.95	98.10	113.94

${\sigma }_{e}^{2}$ error variance, ${\sigma }_{g}^{2}$ genotypic variance, ${\sigma }_{p}^{2}$ phenotypic variance, ${h}_{b}^{2}$ heritability in broad sense, *PCV* phenotypic coefficient of variation, *GCV* genotypic coefficient of variation, *RD* relative difference, *GA* genetic advance, *DTE (day)* days to emergence, *D50%F (day)* days to 50% flowering, *DTM (day)* days to maturity, *PH * (cm) plant height, *NB* number of branches per plant, *NS* number of stems per plant, *NP* number of petioles per plant, *NL* number of leaves per plant, *NNS* no. of nodes per stem, *IL (cm)* inter nodes length, *BFW(g)* biomass fresh weight per plant,* BDW(g)* biomass dry weight per plant, *TNP* total no. of pods per plant, *NMP* number of mature pods per plant, *NIP* number of immature pods per plant, *FPW(g)* fresh pods weight, *DPW(g)* dry pods weight, *PL(mm)* pod length, *PW (mm)* PW, *NSP* number of seeds per plant, *DSW(g)* dry seed weight per plant, *SL(mm)* seed length, *SW (mm)* seed width, *HSW(g)* hundred seed weight, *Shel%* shelling percent ,* HI (%)* harvest index, *Yld* yield (kg ha^−1^).

The relative difference (RD) is the ratio of GCV in association with the respective PCV and the estimated RD values varied from 0.22% (fresh pods weight) to 57.46% for plant height (Table 7). Relatively low difference value between GCV and PCV was recorded for the traits like DTM(9.74%), NB(2.59%), BFW(1.17%), BDW(5.18%), TNP(4.91%), FPW(0.22%), DPW(0.95%), PL(3.15%), DSW(0.57%), HSW(4.71%), and yield (0.95%) kg ha^−1^ and noticed that the variation present among the traits due to the effect of gene and have a better response to direct selection. On the other hand, the traits with higher difference in between their PCV and GCV values indicated the wider genetic variability due to environmental effect and not better feedback to direct selection for the improvement of traits.

The values of heritability in broad sense were observed high for most of the traits evaluated (Table 7) which ranged from 18.09% (PH) to 99.55% (FPW). Very high (≥ 60%) heritability was measured for traits like DTM (81.47%), NB (94.89%), BFW (97.68%), BDW(89.91%), TNP(90.42%), PL(93.79%), DPW(98.10%), DSW(98.86%), HSW (90.80%), HI(83.93%) and yield (98.10%) is the indication of limited chance of environmental effect. The heritability value 30% to 60% was marked for the traits like D50%F(30.32%), PW (56.64%), Shel% (55.22%) and SL(32.25%) which indicate the traits are moderately heritable whereas the trait PH (18.09%) and SW (28.71%) showed heritability below 30% i.e. low heritability. The trait dry seed weight (122.01%) had topmost genetic advance (as percentage mean) value (≥ 20%) or genetic gain while the lowest had (3.97%) for plant hight (PH) (Table 7). Moreover, the higher genetic gain was recorded for the traits like grain yield (113.94%), DPW (113.94%), BDW (106.66%), FPW (93.54%) and BFW (88.82%) with high heritability. In our study, most of the yield contributing traits showed moderate to high heritability with genetic advance (≥ 20%) excluding the traits like SW (GA 8.05%) and Shel% (GA 6.88%) which was ≤ 10%. Consideration of higher value of genotypic coefficient of variation along with higher heritability and genetic advance is the powerful tools of selection for crop improvement than the consideration of individual genetic matrix or measuring unit. However, these traits were governed by additive genes having limited response to environment and suggestively notable for the selection procedure.

### Clustering patterns

**Figure 6:**
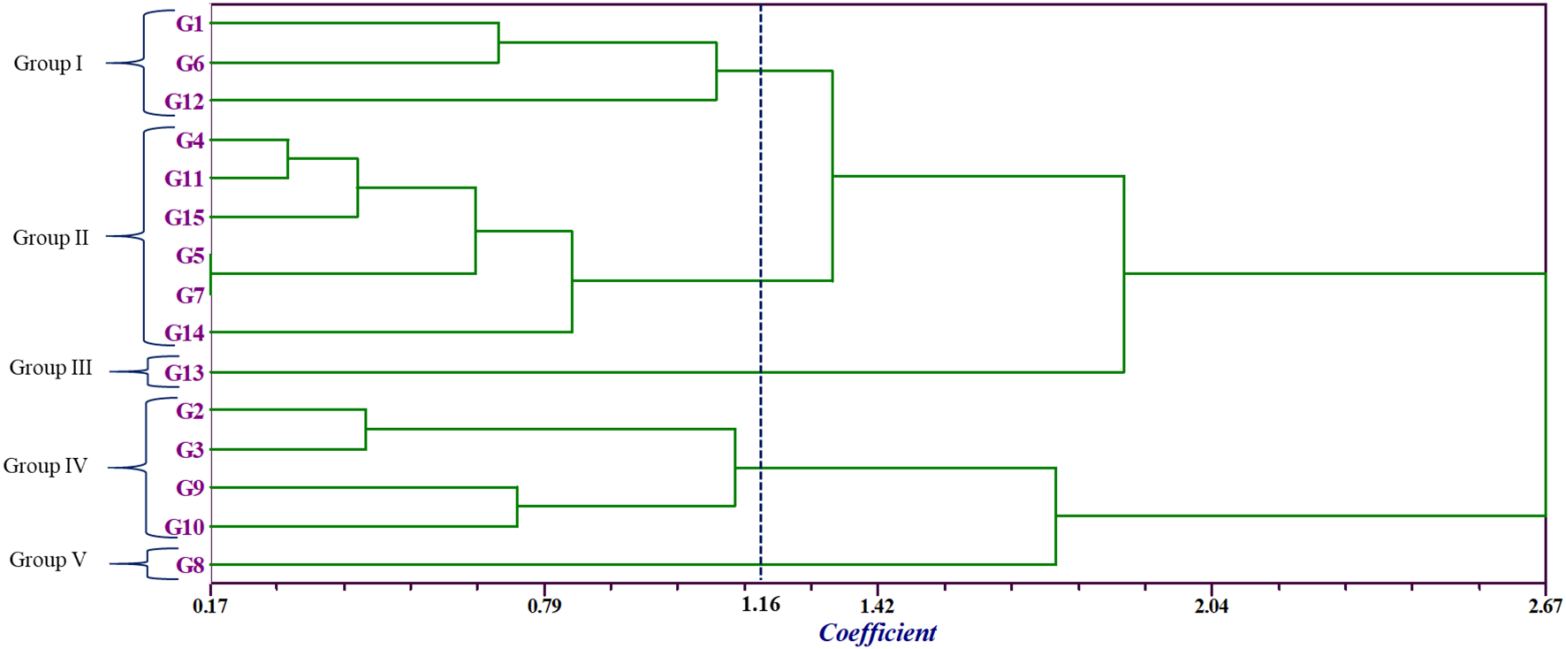
Dendrogram showing the relationship among the Bambara groundnut landraces revealed by non-overlapping (SHAN) UPGMA method.

In this study, the homogenized data was used to calculate the Euclidean distances among the 15 Bambara groundnut accessions and a UPGMA dendrogram was designed (Fig. 6). To discriminate against the relations in the population, the dendrograms of the 15 Bambara groundnut accessions were clustered into five major groups based on their twenty-seven measurable traits at 1.16 dissimilarity coefficients. In the dendrogram, there was a cut off at the point of 1.16 coefficient for ease of interpretation. The Table 8 showed the mean performances of the selections according to each class. Group I represented by G1. G6 and G12 are characterized by early germinating (6 days) but need more time to flowering close to 44 days among the group also took medium time to maturity and plant height (24.45 cm). Internode length was maximum while hundred seed weight and yield kg/ha showed medium values within the five groups. Group II is formed by maximum number of (G4, G5, G7, G11, G14 and G15) accessions characterized by the following traits: maximum maturation date (137 days) and minimum pod length (3.37 mm) with compared to other groups. Group III was illustrated by only one accession (G13). This accession was distinguished by the following traits with minimum values like plant height, branches number, stem number, leaves number, nodes number, internode length, biomass fresh & dry weight, total pod number, no. of mature pod, dry & fresh pod weight, pod width, seed number, dry seed weight, seed length & width, hundred seed weight, shelling percent, yield kg/ha as their most isolated characters. Group IV comprised of four accession G2, G3, G9 and G10 with maximum values of traits like plant height (cm), total pod number, no. of immature pod, dry & fresh pod weight, pod length & width, seed number, dry seed weight, seed length & width, hundred seed weight, harvest index and yield kg/ha as their most special characters. All the accessions under this group had mean yield maximum 2 ton/ha which was due to maximum value of yield contributing traits. The last Group V captured only one accession G8 seemed to have long time to emergence (9 days) but gave early flower with maximum values of traits like branches number, stem number, nodes number, biomass fresh & dry weight, mature pod number, pod length, hundred seed weight and shelling percent as their most distinctive characters. Group IV gave the highest yield (36.48%), while Group III gave the lowest yield (10.71%). Two groups I (19.44%) and group V (18.67%) had accessions near to each other on the aspect of yield traits (Table 8). Moreover, we estimated 70.07% higher ( +) mean yield over the average grand mean yield (1180 kg ha^−1^) for cluster IV whereas the cluster I (9.34%), cluster II (31.44%), cluster III (50.08%) and cluster V ( 12.95%) produced lower (−) yield.

**Table 8:** Mean values of quantitative traits for 5 groups revealed by cluster analysis.

Trait	Cluster				
	Group I	Group II	Group III	Group IV	Group V
	G1. G6, G12	G4, G5, G7, G11, G14, G15	G13	G2, G3, G9, G10	G8
DTE	6.44	8.78	8.00	7.58	9.00
D50%F	43.78	38.94	42.33	36.83	34.33
DTM	128.89	137.56	128.00	127.75	122.33
PH	24.45	23.98	21.57	25.35	25.23
NB	39.78	30.06	23.67	35.58	56.67
NS	13.11	12.44	12.33	14.33	16.67
NP	298.78	326.61	402.00	261.75	165.00
NL	896.33	979.83	1206.00	785.25	495.00
NNS	11.89	11.39	9.00	12.17	12.33
IL	3.37	3.09	2.77	3.14	3.30
BFW	403.06	263.24	109.97	335.78	511.00
BDW	163.72	122.14	50.22	160.84	197.68
TNP	66.22	59.22	51.67	82.00	81.33
NMP	50.22	44.67	35.00	63.50	67.67
NIP	16.00	14.56	16.67	18.50	13.67
FPW	229.57	223.70	155.50	410.95	391.64
DPW	128.37	97.07	70.68	240.79	123.25
PL	24.55	23.96	24.11	34.78	31.56
PW	13.17	12.39	11.03	14.65	14.10
NSP	64.72	57.72	50.17	80.50	79.83
DSW	92.50	68.71	50.15	176.28	92.05
SL	13.29	12.52	11.03	14.83	13.75
SW	9.31	9.01	6.65	9.51	9.32
HSW	209.07	202.92	198.35	274.73	278.26
Shel%	71.50	71.30	71.01	72.24	74.67
HI	43.94	45.63	58.58	63.41	39.07
Yld	1069.71	808.92	588.98	2006.60	1027.09
RPMY (%)	19.44	14.7	10.71	36.48	18.67
RPGY (%)	(−) 9.34	(−) 31.44	(−) 50.08	( +) 70.05	(−) 12.95

*RPMY* relative proportion (%) of mean yield, grand average yield = 1180 kg ha^−1^; *RPGY (%)* relative proportion of grand average yield, ‘( +)’ = yield higher; ‘(−)’ = yield lower. *DTE (day)* days to emergence, *D50%F (day)* days to 50% flowering, *DTM (day)* days to maturity, *PH* plant height (cm), *NB* number of branches per plant, *NS* number of stems per plant, *NP* number of petioles per plant, *NL* number of leaves per plant, *NNS* no. of nodes per stem, *IL (cm)* inter nodes length, *BFW(g)* biomass fresh weight per plant, *BDW(g) *biomass dry weight per plant, *TNP* total no. of pods per plant, *NMP* number of mature pods per plant, *NIP* number of immature pods per plant, *FPW(g)* fresh pods weight, *DPW(g)* dry pods weight, *PL(mm)* pod length, *PW (mm)* PW, *NSP* number of seeds per plant, *DSW(g)* dry seed weight per plant, *SL(mm)* seed length, *SW (mm)* seed width, *HSW(g)* hundred seed weight, *Shel%* shelling percent, *HI (%)* harvest index, *Yld* yield (kg ha^−1^).

#### Heatmap analysis for genotypes and agro-morphological traits

**Figure 7:**
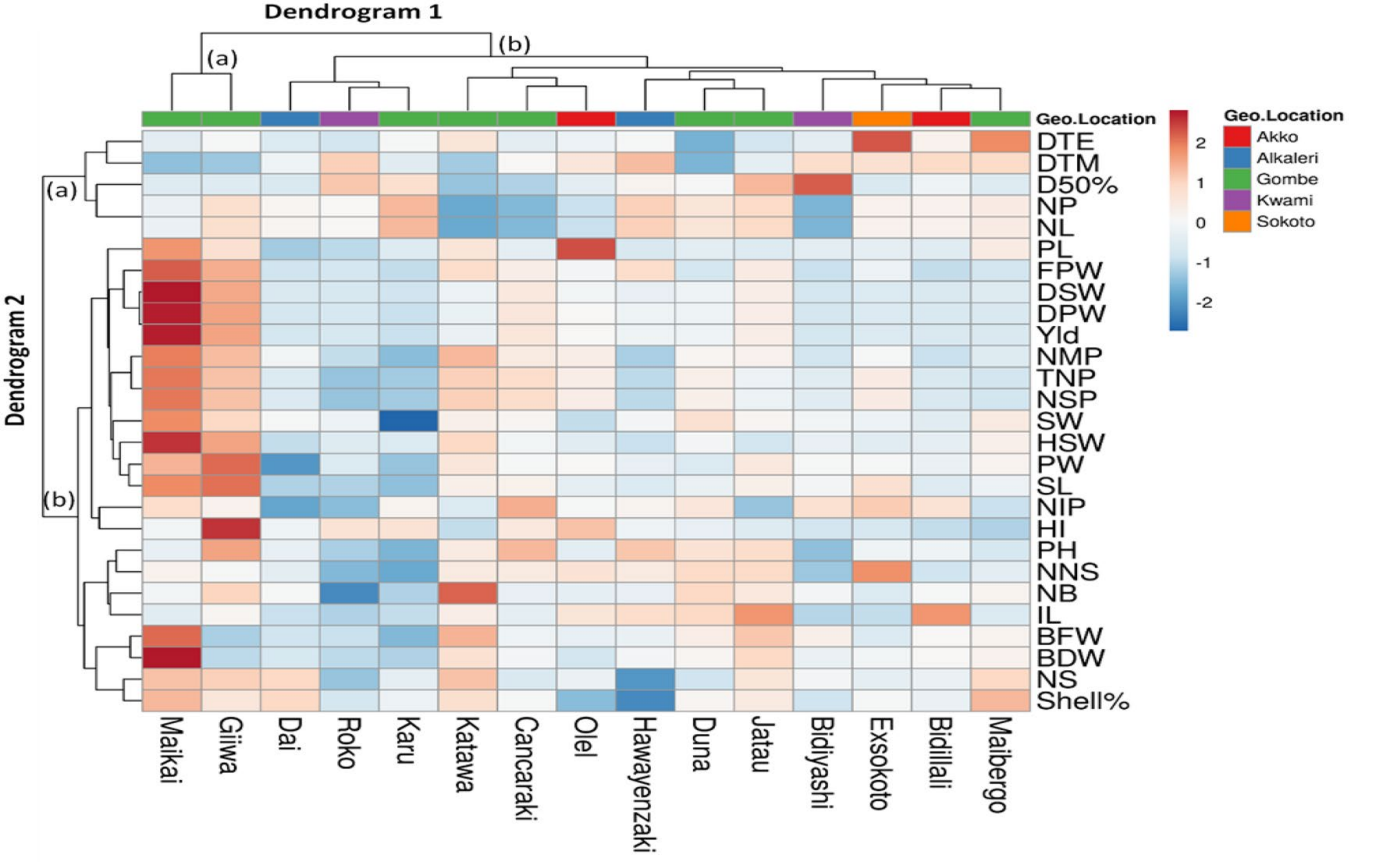
Heatmap and hierarchical clustering (double dendrogram) responses to morphological descriptors of Bambara groundnut landraces constructed using ClustVis tools (https://bio.tools/clustvis). The heatmap plot describes the relative abundance of each Bambara groundnut genotypes (columns) within each feature (rows). The color code (blue to dark red) displays the row z-score: red color indicates high abundance, blue color low abundance. The dendrogram shows hierarchical clustering of Bambara groundnut genotypes based on the Euclidian as the measure of distance and Ward’s cluster agglomeration method.

A heatmap is a data imagining practice that displays extent of a phenomenon as color in two dimensions. The variation in color may be by hue or intensity, giving noticeable visual indications to the reader about how the phenomenon is clustered or varies over space. It visualizes the relative patterns of high-abundance features against a background of features that are mostly low-abundance or absent. Heatmap analysis of agro-morphological descriptors were carried out to show a chromatic evaluation of the Bambara groundnut genotypes. The heatmap analysis constructed double dendrograms, the 1st dendrogram on the vertical direction, an arrangement that represent the Bambara groundnut accessions, and the 2nd dendrogram on the horizontal direction representing traits that influenced this diffusion. Dendrogram 1 showed two major group, group (a) linked to two genotypes Maikai (G2) and Giiwa (G9) while the group (b) comprised of rest 13 genotypes (Fig. 7). Dendrogram 2 also displayed two major groups, group (a) linked to traits DTE, DTM, D50%F and NP whereas other 23 traits belong to group (b). The genotype Maikai and Giiwa under left side of the dendrogram 1 appear in the same cluster having higher values of yield, FPW, DPW, HSW, TNP, NMP, SL,SW, PL, PW, BFW and BDW which separate this two genotype from other genotypes. On the right side of the dendrogram 1, two sub-groups were documented, 1st on the left included three genotypes (Karu, Roko & Dai), which exposes in the lower values for all traits evaluated and similar values were recorded for Hawayenzekai and Duna in right side sub-group of cluster (b). The higher values were recorded for genotype Katawa (NB, BDW, BFW), Jatau (IL & D50%F), Bidiyashi (D50%F), Exsokoto (NNS & DTE), Bidillali (IL) and Maibergo (NS, Shell% & DTE), all other traits exhibits lower values are responsible for constructing different sub-groups among the genotypes. Interestingly, the groups and sub-groups in dendrogram 2 sharply highlighted the discrepancy effects of the diverse the Bambara groundnut landraces.

### Valuation of principal component analysis

**Figure 8:**
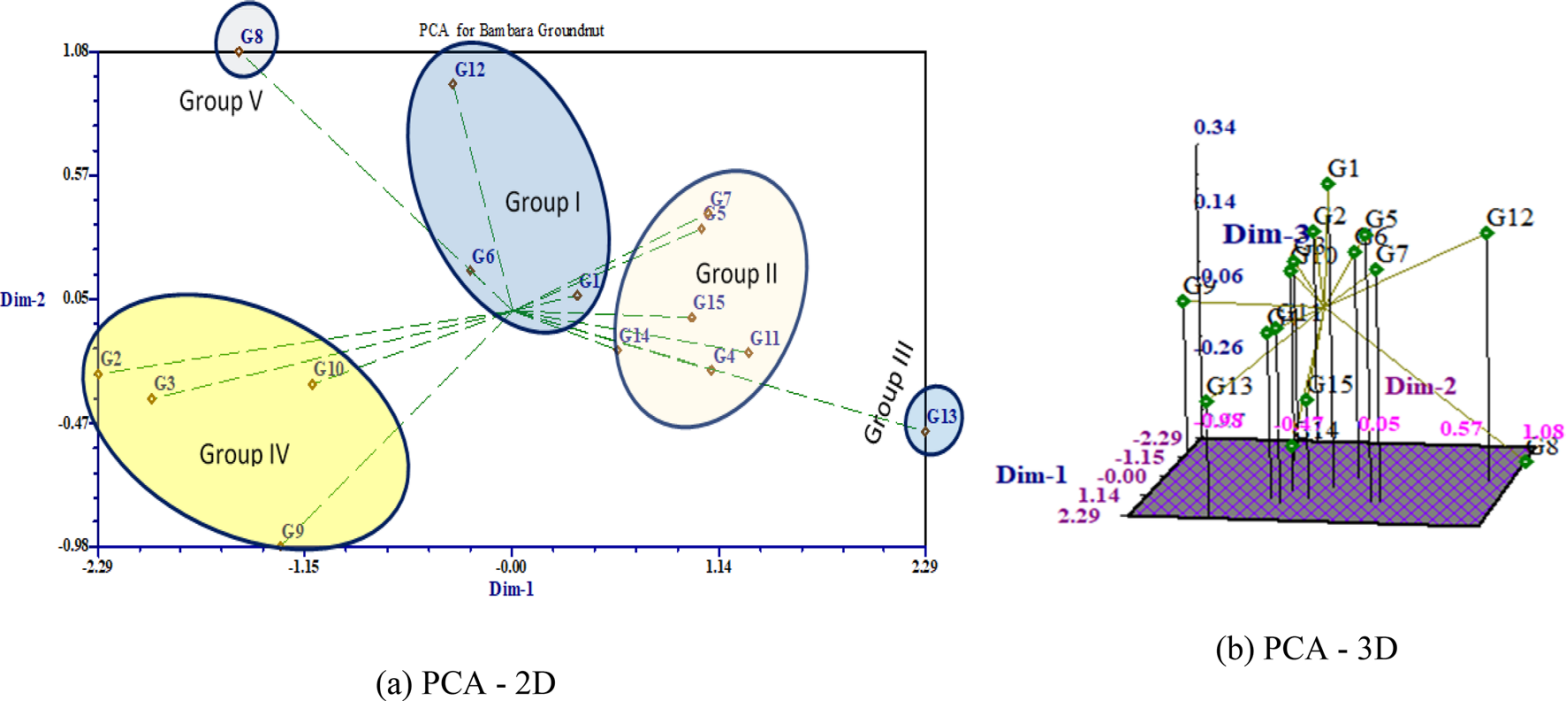
PCA-2D **(a)** and PCA-3D **(b)** graphical relationship among the Bambara groundnuts accessions based on Euclidian distance.

**Figure 9:**
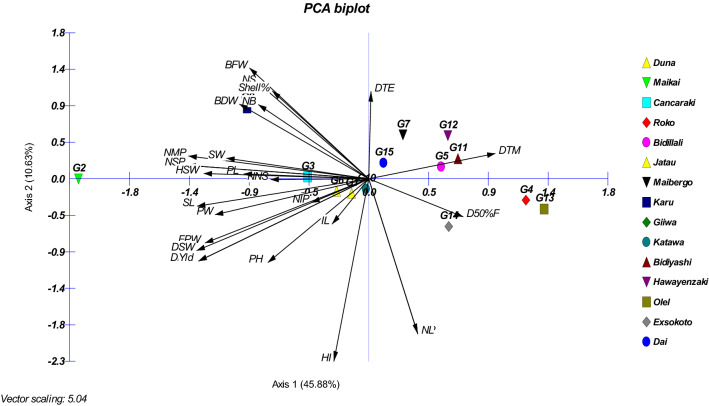
PCA biplot with variables (27) and cases (15) loading of Bambara groundnuts accessions using MVSP.

Based on the results from Table 9 it appeared that the principal component 1 (PC1) accounted for close to 45.88% of the total variation and the characters responsible for genotypes separation along this axis were TNP (highest 0.271), NMP, FPW, DPW, NSP, DSW, SL, HSW and YLD (kg ha^−1^) with high and positive value of coefficient of variation. The second principal component (PC2) associated with the traits D50%F, PH, NP, NL, FPW, DPW, DSW, HI (maximum 0.447) and YLD (kg ha^−1^) accounted for 10.64% of the total variation. About 8.78% of the total variation was detected for Principal Component 3 (PC3) and displayed differences based on PH, NB, NS, NP, NL, NNS, IL (largest 0.472), BFW and BDW. The principal component 4 (PC4) accounted for 8.19% of the total variation and consisted mostly of the traits of D50%F, NS, NP, NL, HSW, and Shell% (maximum 0.444). The variation (6.75%) was found for principal component 5 (PC5) and comprised with D50%F (maximum 0.479), DTM, DSW and YLD (kg ha^−1^). The last principal component 6 (PC6) accounted for 5.69% of the total variation and consisted of the traits DTE, DTM, NP, NL, BDW, NIP, PW and SL up to this principal component covered close to 86% of the cumulative variation. The two-dimensional (Fig. 8a) and three dimensional (Fig. 8b) graphical elucidation demonstrated that most of the accessions were dispersed at low distances whereas the few were dispersed at high distances as reflected by eigenvector (Table 9). The farthest accession from the centroid was G2, G3, G8, G9, G12 and G13 whereas other accessions were near to centroid. The proportion of variation for principal component (PC1) and (PC2) were 45.88% and 10.64% respectively, in which the first principal component occupied the topmost position of the total variation existed (Table 9). PCA biplot loaded the both variables and cases (accessions) at the same time shows how strongly each trait influences a PC and correlated to each other it also shows the how distances the genotype from each other. The lesser angle between two vectors (Fig. 9) indicate higher and positive correlation (e.g. DSW & Yield), when angle between two vectors form 90° indicate no correlation while it goes more than 90° to near 180°, indicate negative correlation between the traits (e.g. HSW & NL) .

**Table 9:** Eigenvectors and values for the first six principal component axes for 27 agronomic traits associated Bambara groundnut accessions.

Character	Eigenvector						Hˊindex (H)	H_max_ = ln(N)	Evenness (E_H_) = H/H_max_
	PC1	PC2	PC3	PC4	PC5	PC6			
Eigenvalue	12.39	2.87	2.37	2.21	1.82	1.54			
Proportion of variance (%)	45.88	10.64	8.78	8.19	6.75	5.69			
Cumulative proportion of variance (%)	45.88	56.51	65.29	73.48	80.24	85.93			
DTE	− 0.003	− 0.214	− 0.072	− 0.044	− 0.407	0.579	2.69	2.71	0.99
D50%F	− 0.141	0.093	− 0.021	0.11	0.479	0.009	2.70	2.71	1.00
DTM	− 0.189	− 0.062	− 0.086	− 0.243	0.077	0.350	2.71	2.71	1.00
PH	0.151	0.205	0.31	− 0.256	− 0.193	− 0.135	2.71	2.71	1.00
NB	0.165	− 0.181	0.299	− 0.022	− 0.194	− 0.249	2.67	2.71	0.99
NS	0.165	− 0.234	− 0.017	0.376	− 0.171	− 0.060	2.69	2.71	0.99
NP	− 0.073	0.379	0.287	0.278	− 0.091	0.270	2.67	2.71	0.99
NL	− 0.073	0.379	0.287	0.278	− 0.091	0.270	2.67	2.71	0.99
NNS	0.146	0.002	0.273	− 0.318	− 0.222	0.101	2.70	2.71	1.00
IL	0.055	0.111	0.472	− 0.217	0.079	− 0.117	2.70	2.71	1.00
BFW	0.178	− 0.271	0.215	− 0.028	0.37	0.053	2.62	2.71	0.97
BDW	0.193	− 0.183	0.193	0.042	0.334	0.230	2.58	2.71	0.95
TNP	0.271	− 0.033	− 0.084	− 0.092	− 0.075	− 0.076	2.69	2.71	0.99
NMP	0.269	− 0.056	− 0.032	0.026	− 0.095	− 0.150	2.68	2.71	0.99
NIP	0.085	0.058	− 0.178	− 0.377	0.036	0.195	2.68	2.71	0.99
FPW	0.244	0.157	− 0.007	− 0.056	0.059	0.087	2.62	2.71	0.97
DPW	0.254	0.201	− 0.072	0.05	0.148	0.043	2.58	2.71	0.95
PL	0.186	− 0.012	− 0.214	− 0.087	− 0.03	0.055	2.68	2.71	0.99
PW	0.229	0.088	− 0.064	− 0.049	0.031	0.198	2.70	2.71	1.00
NSP	0.271	− 0.033	− 0.084	− 0.092	− 0.075	− 0.076	2.69	2.71	0.99
DSW	0.257	0.176	− 0.068	0.09	0.143	0.050	2.57	2.71	0.95
SL	0.256	0.067	− 0.058	− 0.018	− 0.045	0.188	2.70	2.71	1.00
SW	0.212	− 0.05	0.13	0.05	0.13	0.056	2.70	2.71	1.00
HSW	0.246	− 0.013	− 0.165	0.171	0.036	0.088	2.69	2.71	0.99
Shel%	0.144	− 0.215	0.079	0.444	− 0.182	0.015	2.71	2.71	1.00
HI	0.052	0.447	− 0.302	0.024	− 0.175	− 0.242	2.68	2.71	0.99
Yld	0.254	0.201	− 0.072	0.05	0.148	0.043	2.58	2.71	0.95

*DTE (day)* days to emergence, *D50%F (day)* days to 50% flowering, *DTM (day)* days to maturity, *PH * plant height (cm), *NB* number of branches per plant, *NS* number of stems per plant, *NP* number of petioles per plant, *NL* number of leaves per plant, *NNS* no. of nodes per stem, *IL (cm)* inter nodes length, *BFW(g)* biomass fresh weight per plant, *BDW(g)* biomass dry weight per plant, *TNP* total no. of pods per plant, *NMP* number of mature pods per plant, *NIP* number of immature pods per plant, *FPW(g)* fresh pods weight, *DPW(g)* dry pods weight, *PL(mm)* pod length, *PW (mm)* PW, *NSP* no. of seeds per plant, *DSW(g)* dry seed weight per plant, *SL(mm)* seed length, *SW (mm)* seed width, *HSW(g)* hundred seed weight, *Shel%* shelling percent,* HI (%) *harvest index, *Yld* yield (kg ha^−1^) ; *H* Shannon diversity, *N* no. of observations.

### Valuation of Shannon–Weaver diversity (H’ Index)

The Shannon–Weaver diversity index was used to assess the phenotypic diversity for each trait. The estimation of the Shannon–Weaver diversity index (H) and Evenness (E_H_) for the twenty-seven traits shown in Table 9. The Shannon–Weaver diversity index ranged from 2.57 for dry seed weight per plant to 2.71 for plant height, maturity date and shelling percent including the traits like fifty percent flowering date, nodes number per stem, internode length, pod width, seed length and seed width which indicated that maximum diversity (H = 1.70) was present among these traits. The equitability or evenness was found varied from 0.95 to 1.00. Similarly, maximum (E_H_ = 1.00 ) values of evenness was marked for the traits like fifty percent flowering date, maturity date, plant height, nodes number per stem, internode length, pod width, seed width, shelling percent whereas minimum (E_H_ = 0.95) was for biomass dry weight, dry pod weight, dry seed weight, yield kg ha^−1^.

### Selection of Bambara groundnut accessions based on Rank summation index (RSI)

Pearson's correlation coefficients were estimated for all traits to define the traits of a positive or negative association with yield. The traits which were strongly positively associated with yield were used to compute Rank Summation Index (RSI) to lead high yielding Bambara groundnut selection from the population. Furthermore, correlations within a couple of traits are probably significant when the ideal values of the coefficient (%) are higher than 0.20^43^. Using Mulumba and Mock^44^ reported formula of RSI method the accessions were first ranked using average values of the respective traits of positively correlated with yield (here, 1 = topmost and 15 = lowermost), consequently the ranked values of traits were summed to estimate the total performance of individual accessions. In this way, the accession with the lowest RSI score indicates the superior yielding potential. The values of RSI ranking of Bambara groundnut with the traits of positive and strong significantly correlated traits with yield namely fresh pod weight, dry pod weight, no. of seed per plant and dry seed weight per plant are represented in Table 10. Based on yield, the topmost genotype G2 (2991 kg ha^−1^), G9 (2226 kg ha^−1^), G3 (1557 kg ha^−1^), G6 (1414 kg ha^−1^), G10 (1250 kg ha^−1^), G1(1101 kg ha^−1^) were identified as high yielding accessions among the population evaluated with lowest RSI values 4, 8, 16, 21, 23, and 28 were observed, respectively while the entries G13 had the lowest yield (558 kg ha^−1^) with highest RSI values of 59.

**Table 10:** Mean value of positive, strongly significant correlated traits with yield and their rank summation index (RSI).

Genotype	FPW	rsi	DPW	rsi	NSP	rsi	DSW	rsi	RSI = ∑rsi	Yld
G2	568.56	1	359.01	1	92	1	274.96	1	4	2991.77
G9	469.11	2	267.16	2	82	2	197.12	2	8	2226.30
G3	325.06	6	186.91	3	77	4	134.47	3	16	1557.61
G6	332.98	5	169.76	4	63	8	124.72	4	21	1414.67
G10	281.09	7	150.09	5	70	6	98.56	5	23	1250.73
G1	188.59	9	132.21	6	70	7	95.68	6	28	1101.72
G14	388.44	4	130.58	7	53	13	83.35	8	32	1088.15
G8	391.64	3	123.25	8	79	3	92.05	7	21	1027.09
G15	254.63	8	94.38	9	71	5	67.93	10	32	786.47
G7	180.95	11	93.54	10	56	12	71.71	9	42	779.46
G5	157.18	14	92.02	11	58	11	65.26	11	47	766.81
G4	186.22	10	88.69	12	49	15	61.37	13	50	739.05
G11	174.76	12	83.23	13	58	10	62.61	12	47	693.55
G12	167.14	13	83.13	14	60	9	57.10	14	50	692.75
G13	155.50	15	70.68	15	50	14	50.15	15	59	588.98

*FPW (g)* fresh pod weight, *DPW (g)* dry pod weight, *NSP* no. of seed per plant, *DSW (g)* dry seed weight, *Yld (kg/ha)* yield, *RSI *Rank summation index.

## Discussion

### Qualitative disparity

The existence of a significant qualitative variation was found for all the qualitative traits, supported by Gbaguidi et al*.*^45^ he found significant variation among all the qualitative traits. We recorded three types of growth habit and similar observation were noticed by Ntundu et al*.*^1^ in Tanzania, Khan et al*.*^23^ in Malaysia and Azam-Ali et al*.*^46^ in Cameroon. We categorized the vegetative growth of Bambara groundnut namely, bunches type, semi bunches type and spreading type which is matched by the result of Doku^47^ and highly significant difference among the qualitative trait was noted by Egbadzor et al.^48^.

### Quantitative traits

The estimated 27 quantitative traits showed a massive genetic variation and similar variation was confirmed by Ntundu et al*.*^1^ and Aliyu et al*.*^49^ in *Vigna subterranea* (L.) Verdc and the cowpea (*Vigna unguiculata* L)^50^. The estimated high coefficients of variation (CV) in our study is the indication of vast scale of heterogeneity confirmed by Goli et al*.*^51^ in Bambara groundnut. We found D50%F close to 39 days but^52^ noticed close to 68 days in Ghana. The indeterminate^53^ nature of flower bearing make it vital issue for adjustment mechanism to an environment^54^. The inconsistency of flowering time was reported by^51^ from 38 to 68 days; Massawe et al.^10^ from 64 to 76 days; Masindeni^55^ reported 43–80 days and Ouedraogo et al*.*^8^ from 32 to 53 days. Several climatic issues photoperiod, temperature, altitude and soil structure as well as genotypic nature is responsible to bearing flower in Bambara groundnut^28^ and reported flowering happened between 36 to 53 days. In our study, genotype G1, G2, G3, G8, G9 and G10 identified as early flowering lines; early flowering ensures early maturity^56^. A significant difference (P ≤ 0.01) was recorded for maturity (119.67 to 141.33) days is supported by Goli et al*.*^51^ and Masindeni^55^ and due to diverse cultivar along with multi-environmental factors maturation time varied from 90 to180 days^57^. Plant hight had no significant variation, supported by Ntundu et al*.*^1^ in Tanzania and Shegro et al*.*^28^ in south Africa. The yield and yield related traits like TNP, NSP, FPW, DPW, DSW, PL, PW, NMP, NIP and HSW showed high genetic discrepancy, similar variation stated by Shegro et al*.*^28^ with a recommendation of variation happened due to effect of genotype by environment interaction Bambara yield. Hundred seed weight varied from 177.52 g to 360.15 g, is a vital factor for the measurement of morphological traits linked to yield^23,52,55,58^ it also influences the yield directly. The yield of Bambara groundnut was recorded from 146.6 to 2678.6 kg ha^−1^ by Gbaguidi et al.^45^; average 703.3 kg ha^−1^ by FAO^20^; 1058.8 kg ha^−1^ by Dansi et al*.*^59^ in west Africa whereas we calculated from 588.98 to 2991.77 kg ha^−1^. Typically, FAO^20^ estimated average yield Bambara groundnut is lower than our estimated yield 1180 kg ha^−1^.

### Correlation coefficient

In plant breeding correlation matrix is a prominent approach for the judgement of degree of the association between two or more variables, is supported by Mohammed^52^. For superior genotype’s selection programme consideration of correlation matrix can be a great scale of measurement^60^. Strong and positive significant correlation for total number of pods (TNP) was identified with the traits NMP, DPW, NSP, DSW and Yield this result is consistent with the study of Pranesh et al. ^61^ and Jonah et al*.*^62^. We got moderate and positively high significant association of plant hight (PH) with TNP, NMP, FPW and yield can be proposed the selection based on these traits may be beneficial for yield enhancement of this crop as well as fodder production for animal feeding. Similar recommendation was stated by Mohammed^52^ in Cote d’Ivoire and^63^ in Cameroon. Nankar et al*.*^64^ described correlation network in tomato phenotypic diversity and pattern search plot that was also supportive to my findings. Relative abundance study using area plot and pie chart revealed richness of traits diversity for each genotype. The traits possessing higher extent in both area plot and pie chart indicates that these traits are highly contributed to governing the diversity among the landraces.

### Genetic components

For the selection program variation presents among the traits was taken into consideration which depends on the degree of heritability. To know the projected gain from selection, valuation of genetic advance with heritability can be a significant approach of crop improvement. Various research findings reported that the selection may be effective for a specific trait improvement using available genetic variation with the degree of heritability^29,65^. Consideration of both heritability and genetic advance is more effective over the uniquely use of heritability^66,67^. Like the previous reporters Adebola et al*.*^68^ findings, we disclose higher phenotypic variance values than genotypic variance for all traits, indicates the trait expression govern by the environment. The obtained GCV and PCV value was categorized based on the suggested index of 0%-10% for low, 10–20% for moderate and ≥ 20% for high variation^23,69,70^. Intermediate to strong genetic advance with heritability was found for all yield related traits except seed width and shelling% is the indication of the traits have significant potential in the selection process due to low environmental influences, supported by Meena et al.^71^. The improvement of the traits with low heritability and genetic advance can be boost over heterosis breeding this is supported by Usman et al*.*^70^. The value of relative differences between GCV and PCV had higher for the trait plant hight, seed width, days to 50% flowering, and seed length is the sign of higher environmental effect and the improvement of these traits are tough via direct selection whereas the trait with lower difference is the symbol of lower influence by the environment which may give desirable strong and significant output in crop improvement program, is supported by Umar et al.^29^ and Usman et al*.*^70^. Direct selection can be effective considering the traits having low relative differences^72^. Considering the heritability and genetic advance index^23,37^ like as more than 60% for high, 30–60% for moderate, and 0–30% for low, we found the traits BFW (Hb = 97.68% GA = 88.82%), BDW(Hb = 89.91% GA = 106.66%), FPW (Hb = 99.55% GA = 93.54%), DPW(Hb = 98.10% GA = 113.94%), DSW (Hb = 98.42% GA = 122.01%) and yield (Hb = 98.10% GA = 113.94%) were highly heritable together with high genetic advance value, recommended that for crop improvement direct selection can be effective based on these traits with effect of additive genes; similar findings documented by the previous researchers^65,73^. Low to moderate heritability and genetic advance values may hindrance in the trait’s betterment due to high environmental effects over the genetic effects on its stated by Ridzuan et al*.*^74^. So, only an effective selection can be gained picking the traits with higher GCV, PCV, HB, and GA meaning that effect of additive genes is sufficiently robust than environmental effect^70^.

### Clustering patterns

Five clusters were constructed based on the 27 quantitative traits at 1.16 of the distant coefficients that indicates a degree of diversity among the genotypes. The cluster V considered as potential group of genotypes for the crop betterment associated with high yielding capacity. The findings of previous researchers^30,45,75,76^ stated that they constructed same type of cluster and found significant variation regarding morphological traits in Bambara groundnut. The study of Unigwe et al*.*^30^ explored the four distinct groups of Bambara groundnut genotypes in south Africa using UPGMA model. The timing of flowering duration is a motivational factor for the final yield also play a positive role to the best yield of the group and selection could be effective from this class noted by Tourél et al*.*^77^. Flowering in Bambara groundnut is indeterminate up harvesting stage explained by Kumaga et al*.*^78^. However, early flowering has been considered as a well agronomic trait of crops to quick maturity, uniform yield as well as generally crop production^78^ thus, accessions that have early flowering criteria should be treated as best to production of Bambara groundnut^79^. The groups achieved from the cluster analysis of quantitative characteristics illustrate the performances of Bambara groundnut accession cultivated in Benin would be the future guideline for this crop improvement^22,80^. The clustering and characterization of accessions considering their agro-morphological traits and genetic similarity would be the crucial issue to identification and selection of the best parents for hybridisation^81^. Additionally, cluster IV produced 70.05% higher mean yield than the average grand mean yield of 1180 kg ha^−1^ while the other groups gave lower yield and this finding were supported by Onwubiko et al*.*^73^. Therefore, current research represents significant information to the plant breeders based on their similarity and grouping of accessions through univariate and multivariate methods. The heatmap analysis depicted the depth of correspondence among morphological traits evaluated of Bambara groundnut genotypes and this result was constantly supported by Virga et al*.*^82^

### Principal component analysis

The principal component analysis (PCA) is the re-validation instrument of cluster analysis. To estimate the total variation, exist in a set of characters, PCA is effective noted by Johnson^39^. The first axes (PC1) elucidate utmost portion of total variation in any PCA^83^. In our findings first principal component (PC1) accounted more proportion of variation (45.88%) than PC2 (10.68%). Similar result was identified by Mohammed et al*.*^84^ of total variation at 19% (PC1) and 14% (PC2) in Bambara groundnut. The results of several researchers like Usman et al*.*^70^, Farhad et al*.*^85^ & Maqbool et al*.*^86^ supported our findings. Shegro et al.^28^ grouped the 20 Bambara groundnut accessions by PCA analysis using quantitative traits. For yield improvement the selection PC1 was revealed as the most powerful criterion concluded by the work of Adéoti et al*.*^87^ and Mih et al*.*^88^. In my research total pod numbers, mature pods number, seed number, dry seed weight and yield kg/ha occupied high values in PC1. This finding supported by Stoilova & Pereira^80^ described that the most significant components for yield are the pods number and seeds number per plant. The cluster analysis together with principal component analysis explored the common association among landraces in terms of seed yield and related agronomic traits.

### Shannon diversity index (H) and evenness (E)

Shannon’s diversity index (H) is another index that is generally used to categorize the species diversity in a certain community. Shannon’s diversity index is an account for both richness and evenness present in the species also used for a wide diversity of fields. The estimated H’ Index varied from 2.57 for dry seed weight per plant to 2.71 for plant height, maturity date and shelling percent among the phenotypic traits. In our study the observed diversity index value was more than 2.50 for most of the traits evaluated and highest the value indicates higher diversity, though H’ index ranges typically from 1.5 to 3.5 but rare can be reaches 4.5^89,90^. Olukolu et al.^22^ reported H’ Index of nineteen qualitative traits (0.1 to 0.15) and twenty-eight numerical traits (0.09 to 0.16) of Bambara groundnut that supported our findings. Bonny et al.^76^ evaluated the diversity in qualitative traits of Bambara groundnut landraces of similar findings with our result.

### Selection of high yielding accession using RSI method

The result from correlation studies revealed the traits that have a positive significant correlation with yield also give valuable information in a breeding program for the selection of high yielding Bambara groundnut. Similar findings were concluded by Onwubiko et al*.*^43^ and Ajala et al*.*^91^, he got two high yielding genotypes from 33 accessions using the RSI method. Other agronomic evaluations in this study like clustering and principal component analysis also had confirmed the accuracy of the selective index result.

## Conclusion

From the present study an evident has been established that the improvement of Bambara groundnut (*Vigna subterranea* [L.] Verdc.) yield and it related traits can be gained via selection with the valuation of different genetic parameters analysis like GCV, PCV, HB, and GA. Based on the recorded data and considering the supplementary analysis (heatmap study, correlation network, abundance analysis) it can be state that a considerable degree of variation exist in almost all the agronomic traits evaluated in this study. Moderate to perfect significant association was noted between the yield and its related traits. Additionally, this research also depicts selection criteria using the traits which had strongly positive correlation with grain yield. More than 20% PCV and GCV values was estimated for all traits excluding the traits like TNP, PW, NSP, SL, SW, and shell% beside this, the six traits like DSW, DPW, FPW, BDW, BFW, and Grain yield showed high (≥ 20%) genetic advance (as percentage mean) with high heritable values. However, it can be declared that a higher extent of divergence was detected among tested landraces based on H’-index (1.57–2.71) as well as Euclidian distance clustering (into five group). Considering all statistical findings, the genotype G2, G3, G8 and G9 identified as high yielding promising lines and can be use as distance parents for hybridization program. We suggested that further research can be conducted to gain the homogeneity of genotypes based on yield and its contributed traits improvement. Concurrently, we must provide emphasis on intensive research of these potentially high yielding lines, together with conventional breeding and molecular approaches.
